# Iron Transformation Pathways and Redox Micro-Environments in Seafloor Sulfide-Mineral Deposits: Spatially Resolved Fe XAS and δ^57/54^Fe Observations

**DOI:** 10.3389/fmicb.2016.00648

**Published:** 2016-05-10

**Authors:** Brandy M. Toner, Olivier J. Rouxel, Cara M. Santelli, Wolfgang Bach, Katrina J. Edwards

**Affiliations:** ^1^Département des Ressources Physiques et Écosystèmes de Fond de Mer, Water, and Climate, University of Minnesota-Twin CitiesSt. Paul, MN, USA; ^2^Department of Deep-sea Physical Resources and Ecosystems, Centre de Brest, Institut Français de Recherche pour l'Exploitation de la MerPlouzané, France; ^3^Department of Earth Sciences, University of Minnesota-Twin CitiesMinneapolis, MN, USA; ^4^Department of Geosciences and MARUM, University of BremenBremen, Germany; ^5^Department of Biological Sciences, University of Southern CaliforniaLos Angeles, CA, USA

**Keywords:** hydrothermal, East Pacific Rise, X-ray absorption spectroscopy, stable isotopes, micro-environment, mineral alteration, iron, biosignature

## Abstract

Hydrothermal sulfide chimneys located along the global system of oceanic spreading centers are habitats for microbial life during active venting. Hydrothermally extinct, or inactive, sulfide deposits also host microbial communities at globally distributed sites. The main goal of this study is to describe Fe transformation pathways, through precipitation and oxidation-reduction (redox) reactions, and examine transformation products for signatures of biological activity using Fe mineralogy and stable isotope approaches. The study includes active and inactive sulfides from the East Pacific Rise 9°50′N vent field. First, the mineralogy of Fe(III)-bearing precipitates is investigated using microprobe X-ray absorption spectroscopy (μXAS) and X-ray diffraction (μXRD). Second, laser-ablation (LA) and micro-drilling (MD) are used to obtain spatially-resolved Fe stable isotope analysis by multicollector-inductively coupled plasma-mass spectrometry (MC-ICP-MS). Eight Fe-bearing minerals representing three mineralogical classes are present in the samples: oxyhydroxides, secondary phyllosilicates, and sulfides. For Fe oxyhydroxides within chimney walls and layers of Si-rich material, enrichments in both heavy and light Fe isotopes relative to pyrite are observed, yielding a range of δ^57^Fe values up to 6‰. Overall, several pathways for Fe transformation are observed. Pathway 1 is characterized by precipitation of primary sulfide minerals from Fe(II)*aq*-rich fluids in zones of mixing between vent fluids and seawater. Pathway 2 is also consistent with zones of mixing but involves precipitation of sulfide minerals from Fe(II)*aq* generated by Fe(III) reduction. Pathway 3 is direct oxidation of Fe(II) aq from hydrothermal fluids to form Fe(III) precipitates. Finally, Pathway 4 involves oxidative alteration of pre-existing sulfide minerals to form Fe(III). The Fe mineralogy and isotope data do not support or refute a unique biological role in sulfide alteration. The findings reveal a dynamic range of Fe transformation pathways consistent with a continuum of micro-environments having variable redox conditions. These micro-environments likely support redox cycling of Fe and S and are consistent with culture-dependent and -independent assessments of microbial physiology and genetic diversity of hydrothermal sulfide deposits.

## Introduction

Seafloor hydrothermal activity at oceanic spreading centers is one of the fundamental processes controlling the exchange of heat and chemical species between seawater and ocean rocks (Edmond et al., 1979; Stein and Stein, 1994; Elderfield and Schultz, 1996; Wheat et al., 2004). The altered rock and mineral deposits created by hydrothermal circulation are known to harbor microbial communities with ecological and functional characteristics corresponding to the chemistry of the host substrate (Santelli et al., 2008; Orcutt et al., 2011; Sylvan et al., 2012; Lever et al., 2013; Toner et al., 2013). In addition, it has been demonstrated that microorganisms interact with their rock/mineral environment by promoting mineral dissolution and precipitation (Holden and Adams, 2003; Houghton, 2007; Pagé et al., 2008; Templeton et al., 2009; Houghton and Seyfried, 2010). Investigations of the physiological and phylogenetic diversity of rock-hosted prokaryotes using both culture-based and molecular approaches show their important ecological roles in biogeochemical cycles of carbon (C), sulfur (S), nitrogen (N), and iron (Fe) (Reysenbach and Cady, 2001; Slobodkin et al., 2001; Edwards et al., 2003a; Byrne et al., 2009; Yamamoto and Takai, 2011).

The development of microbial habitats within hydrothermal chimney deposits is a combination of physical (temperature, porosity), chemical (dissolved and mineral), and biological factors (biofilms, mineral alteration). It is well-established that steep temperature and geochemical gradients form within the walls of actively venting chimneys (Tivey, 1995). Corresponding changes in microbial communities along physical and chemical gradients have been demonstrated at various levels of spatial resolution (Karl, 1995; Schrenk et al., 2003; Nakagawa et al., 2005; Pagé et al., 2008; Takai et al., 2008; Callac et al., 2015). The development of micro-environments within chimneys could explain the diverse genetic potential and wide range of metabolisms observed in organisms cultured from sulfide deposits. Analytical tools able to measure chemical, mineralogical, and isotopic information on the micron spatial scale are available, and the geoscience community has begun to apply them in concert (Marcus et al., 2015). Through the combination of these analytical approaches, one can now define the properties of micro-environments and gain the information needed to interpret micro-habitats or micro-niches within rock and mineral substrates.

In mid-ocean ridge (MOR) hydrothermal systems, Fe is a fundamental element (Emerson, 2016). Iron deserves special attention when considering the biogeochemistry of mid-ocean ridges because it: (1) is abundant in most vent fluids; (2) has dynamic solubility properties and precipitates with S to form part of the physical structure of sulfide deposits; (3) has dynamic oxidation-reduction (redox) properties and the ability to set and record redox conditions within fluid flow paths; and (4) is a potential substrate for microbial energy (Fe reduction) and respiration (Fe oxidation). In mid-ocean ridge systems, such as the East Pacific Rise at 9–10°N or Juan de Fuca Ridge Main Endeavor Field, the breadth of possible biogeochemical roles for Fe is fully populated. Hydrothermal sulfides are observed as active and inactive chimneys, massive sulfide deposits, and particles in hydrothermal plumes settling from the water column (Feely et al., 1992, 1994; Hannington et al., 1995; Tivey, 2007; Rouxel et al., 2008a; Toner et al., 2009a; Fouquet et al., 2010; Yucel et al., 2011; Breier et al., 2012). Iron (oxyhydr)oxides crusts form through the alteration of Fe-rich basalts and sulfides, and as microbial mats associated with diffuse venting (Alt, 1988; Mills and Elderfield, 1995; Wheat et al., 2000; Boyd and Scott, 2001; Emerson and Moyer, 2002; Edwards et al., 2011). The oxidizing conditions at the seafloor create a driving force for conversion of hydrothermally derived Fe(II)aq, as well as ferrous Fe in basalt and sulfide minerals, to Fe(III)-bearing minerals through chemical and biological means (Feely et al., 1994; Field and Sherrell, 2000; Edwards et al., 2003b; Toner et al., 2009b). In addition to the mineral forms of Fe, complexes with particulate and dissolved organic matter occur (Bennett et al., 2008; Toner et al., 2009a; Breier et al., 2012; Hawkes et al., 2013) and microorganisms take up hydrothermal Fe in plumes by a variety of mechanisms (Li et al., 2014).

Despite our understanding of the potential (bio)geochemical pathways for Fe transformation in hydrothermal systems, the actual Fe pools and most important mechanisms of transformation are difficult to measure and are an area of active research (Saito et al., 2013; Fitzsimmons et al., 2014; Resing et al., 2015). The strategic application of analytical tools over a range of spatial scales, from dissolved Fe species over a 1000 km to particulate Fe species within submicron aggregates, presents a way forward. In this contribution, we investigate the μm- to mm-scale chemistry of active and extinct hydrothermal sulfide deposits using micro-probe X-ray diffraction (μXRD), micro-probe Fe 1s X-ray absorption spectroscopy (μXAS), and spatially resolved Fe stable isotopes (laser ablation and micro-drilling). The main goal of this study is to describe the Fe transformation pathways in natural sulfide mineral deposits at the seafloor, and examine the products of alteration for indications or markers of biological activity. The research is an extension of incubation studies that subjected polished hydrothermal sulfide samples to seafloor conditions over a known period of time (Edwards et al., 2003b; Toner et al., 2009b) and Fe isotope studies of seafloor hydrothermal vents (Rouxel et al., 2004, 2008a). For our study of natural sulfide mineral alteration, we chose the East Pacific Rise (EPR) 9–10°N area because hydrothermally inactive sulfide deposits from this location are known to host microbial communities with the genetic potential to alter Fe- and S-bearing minerals through redox reactions (Sylvan et al., 2012; Toner et al., 2013). Our investigation reveals a suite of complex Fe transformation pathways, each of which can be partial or complete. We find that metastable Fe oxyhydroxide minerals persist in the samples despite strong evidence for dynamic Fe transformations, and that Fe isotopic fractionation creates very light isotopic signatures (δ^57^Fe values as low as –7‰) for Fe oxyhydroxides in some samples but isotopically heavy in others. The most important outcome of this work is a record of micro-environments within active and inactive sulfide deposits. These micro-environments are consistent with the diverse genetic potential, and correspondingly wide range of potential metabolisms, observed in organisms cultured from sulfide deposits. Micro-environments favorable to microbial Fe and S oxidation and reduction are supported by mineral and isotopic signatures. However, we did not identify any uniquely biological signatures and attribute this outcome to the complex interplay between biotic and abiotic reactions.

## Methods and materials

### Sample collection and processing

Hydrothermally active and inactive sulfide samples were collected from chimneys and massive sulfide deposits during an *R/V Atlantis—DSV Alvin* cruise to the East Pacific Rise (EPR) Clipperton and Siquieros Fracture Zones 9°28′N–9°51′N in 2004. On *Alvin* dives 4053, 4057, and 4059, samples rich in Zn and Fe were collected from: (1) K Vent; (2) north of Bio 9 Vent; (3) south east of Bio 9 Vent; and (4) an extinct, off-axis chimney (Figures S1, S2). Seafloor samples were collected in individual bio-boxes attached to Alvin's basket to avoid cross contamination and minimize exposure to surface sea water. Once on board, the samples were stored in the bio-boxes in the 4°C cooler until they were processed in the lab. The samples were placed on sterile Al-foil and sterile tools were used to split subsamples for microbiology and mineralogy studies. Samples for geochemical analysis were allowed to air-dry shipboard and were stored under ambient conditions until embedding and sectioning. Although residual reduced vent fluids in the active chimneys could oxidize during sample transport and handling, the amount of Fe in residual pore water relative to Fe in the overall sample is small. Therefore, transformations of Fe during sample transport and handling are not expected to affect the overall analysis of the samples. The sulfide mineral composition and associated microbial communities for the sample set has been described previously (Rouxel et al., 2008a; Sylvan et al., 2012; Toner et al., 2013). The samples for this study are listed in Table S1.

K Vent is an active sulfide mound hosting spires and relic chimney debris (i.e., rubble). K Vent samples have the unique identifier *EPR-4053-M* and the three samples were: EPR-4053-M3, rubble; EPR-4053-M1-A1, and EPR-4053-M1-A2, both are active spire samples (Figure S3). K Vent sulfides have central conduits of sphalerite (ZnS), marcasite (FeS_2_), and silica-rich materials while the external walls are composed of sphalerite, pyrite (FeS_2_), and silica (Rouxel et al., 2008a). The inactive massive sulfide samples (*EPR-4057-M2* and *EPR-4059-M3*) are from two different locations in the vicinity of Bio 9 Vent. EPR-4057-M2 has a relic central conduit of marcasite with finely disseminated sphalerite and pyrite throughout, and an external wall of marcasite and Fe oxyhydroxides. EPR-4059-M3 also has a marcasite conduit with sphalerite-pyrite core and sphalerite-pyrite lined fossil tube worms. The extinct, off-axis chimney *EPR-4059-M4* lacks a central conduit and has cm-thick sphalerite layers that transition to sphalerite-pyrite and an external wall covered with Fe oxides and silica.

Air-dry rock specimens were embedded in Epo-tek 301 two-part epoxy (Epoxy Technology). Cross-sections were cut with a wafering saw and adhered to standard glass microscope slides for polishing: producing coarsely polished thick sections (Figure S4). One sample, *EPR-4059-M3*, has a micro-probe quality polished section (30 μm; Spectrum Petrographics, Inc.). The polished sections were used for synchrotron radiation X-ray microprobe measurements while thick sections were used for laser-ablation and micro-drilling for isotope analysis.

### Scanning electron microscopy (SEM)

Subsamples of several EPR sulfides were fixed in 4% paraformaldehyde at room temperature for 4 h, rinsed with a 1:1 phosphate buffer solution (PBS), and stored in PBS-ethanol at −20°C shipboard. Sample EPR-4059-M4 was subjected to critical point drying and platinum sputter coating prior to SEM imaging at the University of Minnesota, Characterization Facility with a field emission gun scanning electron microscope (JEOL 6500).

### Synchrotron microprobe X-ray absorption spectroscopy (XAS) analysis

Iron speciation was measured by Fe 1s XAS in fluorescence mode at the hard X-ray microprobe beamline 10.3.2, Advanced Light Source (ALS), Lawrence Berkeley National Laboratory, Berkeley, CA (Manceau et al., 2002; Marcus et al., 2004). The monochromator was calibrated by setting the inflection point of an Fe 1s XAS spectrum, collected from an Fe foil, to 7110.75 eV (Kraft et al., 1996). The distribution of elements in polished sections was determined by micro-focused X-ray fluorescence (μXRF) mapping using a seven-element Ge solid-state fluorescence detector (Canberra). X-ray fluorescence maps at multiple incident energies were collected to determine the distribution of elements, including Si, S, Ca, Mn, Fe, Co, Cu, Ni, Zn, and As. These XRF maps were then dead-time corrected, registered, and combined into a single composite map with custom beam-line software (Marcus et al., 2004). Composite XRF maps were used to locate sample locations for point Fe XAS data collection. XRF map measurements used 3 × 3 μm^2^ beam spot on the sample.

Iron 1s spectra in the X-ray absorption near edge structure (XANES) energy range were used to survey the oxidation state and mineral class of the Fe-bearing phases. Iron 1s spectra in the extended X-ray absorption fine structure (EXAFS) energy range were conducted in selected locations to better describe the type of Fe oxyhydroxide phases present. XANES and EXAFS measurements used 10 × 3 μm^2^ beam spot on the sample. Iron EXAFS data collection to a reciprocal space (k-space, Å^−1^) value of 14.4 was attempted in all cases to provide adequate resolution for second shell Fe-Fe interatomic distances (Combes et al., 1989). However, the final usable EXAFS data range (based on signal to noise quality) extended to 11.75 to 12.6 Å^−1^ and shell-by-shell fitting was not performed. All Fe 1s spectra were dead-time corrected, energy calibrated, averaged, pre-edge subtracted, and post-edge normalized. Iron EXAFS scans were spline subtracted and converted to reciprocal space (Marcus et al., 2004; Webb, 2005). Iron XANES spectra were corrected for over-absorption induced distortion:

$$\begin{array}{l} \mu_{corrected}=\mu_{exp}/\left(1+a\;\left(1-\;\mu_{exp}\right)\right) \end{array}$$

where *a* was adjusted to obtain agreement between the corrected spectrum and high quality reference standards. Iron EXAFS data collection targeted sample locations with low over-absorption induced distortion; therefore, EXAFS spectra were not corrected for the phenomenon.

Iron XANES (43 spectra) and EXAFS (8 spectra) data were subjected to principal component (PCA) and target transformation (TTA) analysis using SixPack and BL10.3.2 software using methods described previously (Manceau et al., 2002; Webb, 2005; Toner et al., 2006; Breier et al., 2012). Subsequently, linear combination fitting (LCF) of experimental spectra was performed using a library of standards (Hansel et al., 2003; Marcus et al., 2008; Toner et al., 2009b) and custom beamline software (Marcus et al., 2004). A summary of the reference materials is presented in Table S2. The biogenic Fe oxyhydroxide reference spectrum was collected from an Fe-encrusted biofilm (composed of twisted-stalk microbial particles) formed on porous chimney sulfide chips during a 2 month incubation near the Juan de Fuca Ridge (Edwards et al., 2003b). Iron EXAFS was used to describe the Fe-precipitates as polymerized Fe(III) with short-range structure characterized by edge-sharing features and little three-dimensional ordering (Toner et al., 2009b). The best LCF was chosen based on the normalized sum square parameter (NSS):

$$\begin{array}{l} NSS=100\times \left[\sum {\left(\mu {\;}_{exp}-\mu {\;}_{fit}\right)}^{2}/\sum {\left(\mu {\;}_{exp}\right)}^{2}\right] \end{array}$$

where the addition of a spectral component to the fit required a 10% or greater improvement in the NSS value. The Fe XAS reference materials deemed most appropriate for this dataset via PCA-TTA are listed in Table S2. The error on the percentages of species present is estimated to be ±10%.

### Synchrotron radiation X-ray diffraction

X-ray diffraction (XRD) patterns were collected for sample *EPR-4053-M2* from a polished thick section at the ALS, Lawrence Berkeley National Laboratory, Berkeley, CA, USA, on beamline 7.3.3 (Tamura et al., 2002). While Fe XANES and EXAFS are effective at distinguishing among mineralogical classes, in certain cases, they do not provide strong distinction among minerals within the same class (O'Day et al., 2004; Prietzel et al., 2007). For this sample, XRD was used to verify Fe XANES and EXAFS observations of the mineral goethite (α-FeOOH). The area of interest for XRD data collection was located using XRF mapping. The XRD patterns were collected with a CCD camera in reflection mode with a 6.3 keV incident energy and 15 × 8.5 μm^2^ spot size at the sample. The diffraction patterns were processed with the software *XMAS* for comparison to standards (Tamura et al., 2003).

### Multi-collector inductively coupled plasma mass spectrometry (MC-ICP-MS)

Fine scale variations of Fe and S isotope compositions of Fe-oxyhydroxides and pyrite across chimney walls were primarily analyzed using laser ablation technique coupled to multicollector inductively coupled plasma mass spectrometry (MC-ICP-MS). Micro-drilling was also used to allow matrix-free Fe-isotope analysis of mixed Fe-Si oxide and sulfide minerals after complete chemical purification following previously developed methods (Rouxel et al., 2005, 2008a).

For coupled Fe- and S-isotope analysis of pyrite, we adapted a technique previously used by (Craddock et al., 2008) for the determination of ^34^S/^32^S isotope ratios in S-bearing minerals. We used a Nd:YAG deep UV (213 nm) laser ablation system (NewWave™ UP213) coupled to MC-ICP-MS (Neptune, Thermo Scientific) operating at the Woods Hole Oceanographic Institution. The use of high resolution sector-field mass spectrometry removes major isobaric interferences on S-isotopes from O^2+^, as already described in numerous studies (Mason et al., 2006; Craddock et al., 2008; Paris et al., 2013). A similar high-resolution approach was used for Fe-isotope analysis (Graham et al., 2004; Horn et al., 2006; Nishizawa et al., 2010). For the simultaneous analysis of both Fe- and S-isotope values in pyrite, our method involved data acquisition in two sequences (i.e., peak jumping mode) allowing the simultaneous determination of ^32^S, ^33^S, ^34^S (sequence 1), and ^52^Cr, ^54^Fe, ^57^Fe (sequence 2). This approach allows determining high-precision ^34^S/^32^S and ^57^Fe/^54^Fe and on-line correction of potential ^54^Cr interference on ^54^Fe using ^52^Cr.

The laser setup was similar to a published method (Craddock et al., 2008): spot diameter of 60 μm, 10 Hz pulse rate, laser intensity 50–70%, energy density 9–10 j/cm^2^. Note that under these conditions, ^56^Fe signal intensity was found to be >50 V in some cases (i.e., maximum signal measurable on the Neptune Faraday cups), precluding the measurement of δ^56^Fe values. For the determination of only Fe-isotope ratios in pyrite and Fe-oxyhydroxide, we also used a peak jumping mode as follows: ^54^Fe, ^56^Fe, ^57^Fe, ^58^Fe+^58^Ni, ^60^Ni, ^61^Ni, ^62^Ni (sequence 1), ^52^Cr (sequence 2), ^32^S (sequence 3), ^28^Si (sequence 4). Using this approach, all Fe isotopes could be determined together with Ni isotopes (^62^Ni/^60^Ni) that are used for mass bias correction. ^52^Cr was also measured to allow minor isobaric correction on ^54^Fe. The measurements of ^32^S and ^28^Si also allowed us to check for the potential presence of silica (e.g., amorphous silica) and pyrite during analysis of Fe-oxyhydroxides. Operating parameters for laser analysis of Fe isotopes were optimized in order to provide the most stable signal intensities during ablation. The laser was operated under the same setup as for coupled S-Fe isotope measurements but with a smaller spot size of 25 μm to obtain a maximum signal on ^56^Fe below 40V.

As discussed in Craddock et al., 2008, a line scan (“raster”) protocol was used in preference to a single crater mode in order to obtain a higher and more uniform rate of material removal with respect to time. The raster mode utilizes a movable sample stage under a fixed laser beam to generate the desired raster pattern. The size of the trench formed during ablation was about 200 μm in cross-sectional area. A scan speed of 5 μm s^−1^ was used during ablation. Total acquisition time was about 4 min and results in ablation of about 15 μg of sample. The signal intensity was monitored to ensure that transport of sample into the ICP-MS does not significantly diminish as material is ablated during analysis.

The laser was connected directly to a PFA scott-type spray chamber (Savillex) via 3 mm internal diameter Tygon tubing and used helium (He) as the carrier gas from the laser to the ICP. The setup was such that laser ablation and solution aspiration could be operated simultaneously to enable laser ablated particles to be efficiently mixed with an ultra-pure 0.28 M HNO_3_ blank solution prior to injection into the ICP torch. Thus, particles were effectively analyzed as a “wet plasma” ensuring that ablated aerosols were closely matrix-matched to solution standards. Since solution and *in situ* laser analysis was performed interchangeably with identical instrumental setup, the correction of instrumental mass bias used a combination of the “sample-standard bracketing technique” as well as internal normalization technique (i.e., Ni isotopes). Here, we used Ni isotope standard NIST 986 of known isotope composition. For coupled Fe- and S-isotope analysis 34S) of pyrite, the data were calibrated against δ^57^Fe and δ (i.e., aqueous standards prepared in appropriate matrix composition (molar S/Fe ratios at 2) and two internal pyrite standards GAV-18 and FL-19-09. The concentrations of Fe and S (i.e., sulfate form) solution prepared from internal standards (Spex Certiprep) in 0.28 M HNO_3_ were adjusted to obtain similar signal intensity of the ablated sample and bracketing standard solution, typically with less than 20% difference g/g of Fe and S). When only Fe-isotope μ (corresponding to about 10 ^57^Fe), bracketing standard solutions δ^56^Fe and δ-values were measured (g/g). During each laser μg/g to 5 μ were prepared at a concentration of two analysis run, a Ni standard solution was introduced continuously in the spray chamber and mixed with sample aerosols to allow internal mass bias corrections as described elsewhere (Poitrasson and Freydier, 2005; Rouxel et al., 2005). An example laser isotopic run is presented in Figure S5.

We have examined and determined rigorous corrections for analytical difficulties such as instrumental mass bias, unresolved isobaric interferences, blanks, and laser ablation- and matrix-induced isotopic fractionation. By comparing raw Fe and S isotope ratios measured on standard minerals and calibrated against standard solutions, it was possible to determine the instrumental mass bias (i.e., isotope fractionation induced by laser ablation). Calibrated against the pure IRMM-14 solution, duplicated δ^57^Fe analysis of GAV-18 pyrite standard yielded 0.48 ± 0.22‰ (2 sd, *n* = 13), which is about 0.17‰ lighter than bulk δ^57^Fe value determined at 0.65 ± 0.14 (2 sd, *n* = 2). As already discussed for S isotopes using similar experimental setup (i.e., raster mode, Craddock et al., 2008), the isotopic compositions of the GAV-18 standard determined with both methods were identical within analytical uncertainties. This indicates that laser ablation introduced isotope fractionations that were within the analytical uncertainties (±0.2‰ at 2 sd).

To further evaluate whether Fe isotope analyses were potentially affected by the presence of S and Si impurities in Fe-oxyhydroxides, we added S and Si at various concentrations to pure standard solutions. The results show that, after appropriate corrections with Ni isotopes, no bias could be detected up to Si and S/Fe ratios (g/g) in solutions up to 0.7 and 2.5, respectively (Figure S6).

Although most advanced femtosecond ablation techniques enable matrix-independent calibration for Fe isotope analysis (Horn et al., 2006), our laser ablation technique may potentially be prone to significant fractionation during ablation and matrix effects that affect our accuracy and precision. Hence, we compared the Fe-isotope data obtained by laser ablation MC-ICP-MS with Fe-isotope data acquired through conventional MC-ICPMS technique after microdrilling and complete chromatographic purification. We collected powder micro-samples using a microdrill device (New Wave Micromill equipped with drill bit diameter of 700 μm) across the samples investigated by LA-MC-ICP-MS. Following the same protocols as for the bulk chimney samples (Rouxel et al., 2008a) we digested these micro-samples, separated Fe through anion-exchange chromatography procedure and measured Fe isotopes by MC-ICP-MS (Neptune, Thermo Scientific at Pole Spectrometry Ocean, Brest). The results on standards and comparison between laser-ablation and microdrilling techniques suggest that both approaches yield similar results within analytical uncertainties.

## Results

### K Vent diffuser site

K Vent (Figures S1A,B) is an active diffuser site where active chimney spires (EPR-4053-M1-A1 and EPR-4053-M1-A2; Figures S2A,B) and inactive chimney rubble (EPR-4053-M3; Figure S2C) were collected. In the following paragraphs, for each of these samples, a description of the sample texture, Fe chemistry, and Fe (and in some cases S) isotopic composition is provided.

**Figure 1:**
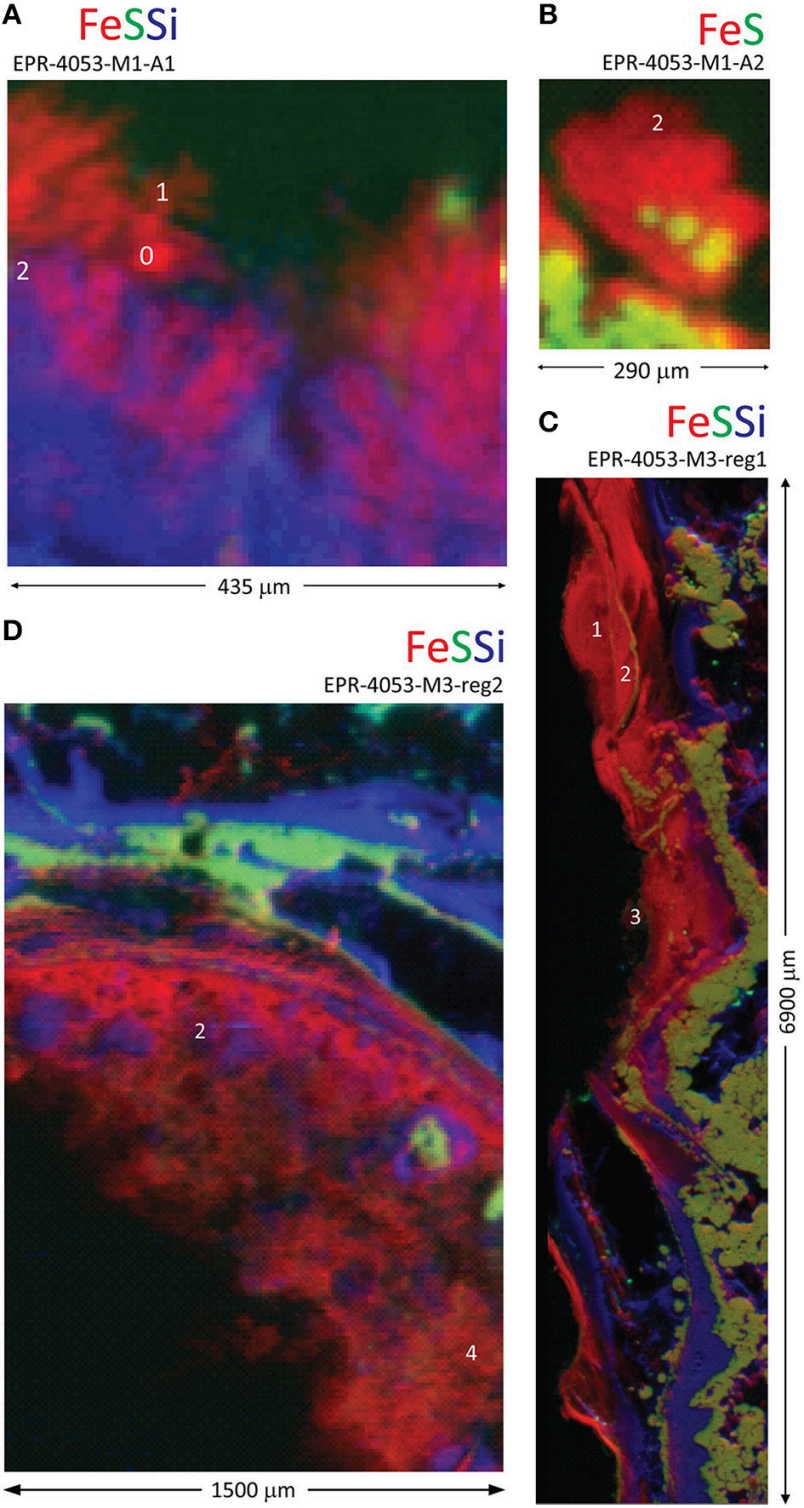
**Distribution of Fe, S, and Si in inactive spire and chimney rubble samples from K Vent**. Red-green-blue (Fe-S-Si) tri-color X-ray fluorescence maps for **(A)** inactive spire (EPR-4053-M1-A1), **(B)** inactive spire (EPR-4053-M1-A2), **(C)** chimney rubble (EPR-4053-M3-reg1), and **(D)** chimney rubble (EPR-4053-M3-reg2). White numbers indicate locations for Fe X-ray absorption near edge structure (XANES; Table S3) or extended X-ray absorption fine structure (EXAFS; Table S4) data collection.

The seawater exposed surface of the actively diffusing spire (EPR-4053-M1-A1) is composed of an Fe-rich and S-depleted zone underlain by a Si-rich layer (Figure 1A). Linear combination fitting (LCF) results for Fe XANES and EXAFS spectra from this sample are consistent with ferrihydrite; 2-line and 6-line [Fe_10_O_14_(OH)_2_ idealized formula (Michel et al., 2007); Tables S3, S4]. The Fe-rich and S-depleted zone in the actively diffusing spire sample EPR-4053-M1-A2 is displayed in Figure 1B). The Fe XANES and EXAFS data for this sample are consistent with an Fe(III)-rich biogenic-like Fe oxyhydroxide phase with ferrihydrite contributions: either 2-line or 6-line satisfies the LCF. Both samples investigated are from the base of the active spire of K Vent (Figure S3).

**Figure 2:**
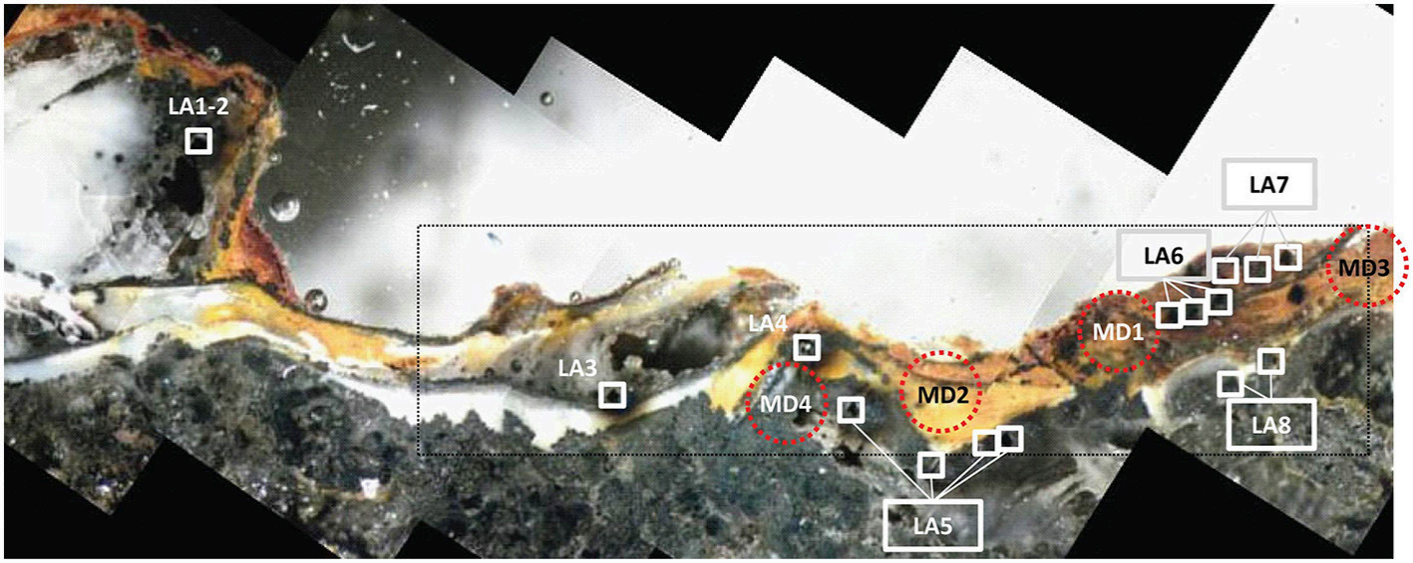
**Light microscope image for K Vent sample EPR-4053-M3-reg1**. The area of the sample shown in Figure 1C is indicated by the black rectangle. Image is annotated with white boxes indicating laser ablation (LA) areas and red dashed circles indicating locations for micro-drilling (MD). Both laser ablation and micro-drilling produced samples for isotopic analysis (Table S5).

Two seawater-exposed surfaces of the K Vent inactive rubble (EPR-4053-M3) are shown in Figures 1C,D. Both regions of the sample are characterized by complex interweaved layers of Fe(III), Si, and sulfide. Petrographic observation and X-ray fluorescence maps (Figure 2) show the co-occurrence of Fe oxyhydroxide, silica (mainly amorphous silica) and pyrite with often rounded and colloform textures. Pyrite spherules (about 50–100 μm diameter) are particularly common along former pyritized worm tubes that are partially filled by silica and Fe oxyhydroxides. The Fe XANES data from both regions indicate that the Fe(III) layers are composed of a biogenic-like Fe oxyhydroxide (40–60 mol%; Table S3) and have varying contributions from goethite (α-FeOOH) or akaganeite (β-FeOOH). Fits to Fe XANES and EXAFS data are in good agreement (Table S4). Locations of coupled Fe and S isotope measurements for chimney wall pyrite, as well as colloform and spherulitic pyrite, using laser ablation are also shown in Figure 2 with results in Table S5. Results show relatively homogenous isotope values, with average δ^57^Fe = −2.0 ± 1.0‰ (2 sd, *n* = 8) and δ^34^S = 3.3 ± 0.9‰ (2 sd, *n* = 8) which is globally consistent with bulk chimney pyrite values (δ^57^Fe = –1.49 ± 0.16‰ and δ^34^S = 1.4 ± 0.2‰) (Rouxel et al., 2008a). Also in EPR-4053-M3-reg1, Fe oxyhyroxides occur in association with minor silica (Figure 2) forming a mm-thick external wall. Spatially resolved analyses using a microdrilling technique reveal a range of δ^57^Fe values from −0.32 ± 0.10‰ (2 sd) down to –0.8 ± 0.14‰ (2 sd) which are similar, albeit slightly heavier, than laser ablation analysis ranging from −0.44 ± 0.14‰ (2 sd) down to −1.35 ± 0.5‰ (2 sd). Compared to colloform pyrite from the outer chimney wall, Fe oxyhydroxides show enrichments in heavy Fe isotopes by up to 2.3‰ (i.e., relative to ^57^Fe/^54^Fe ratios).

### Bio 9 massive sulfide deposits

**Figure 3:**
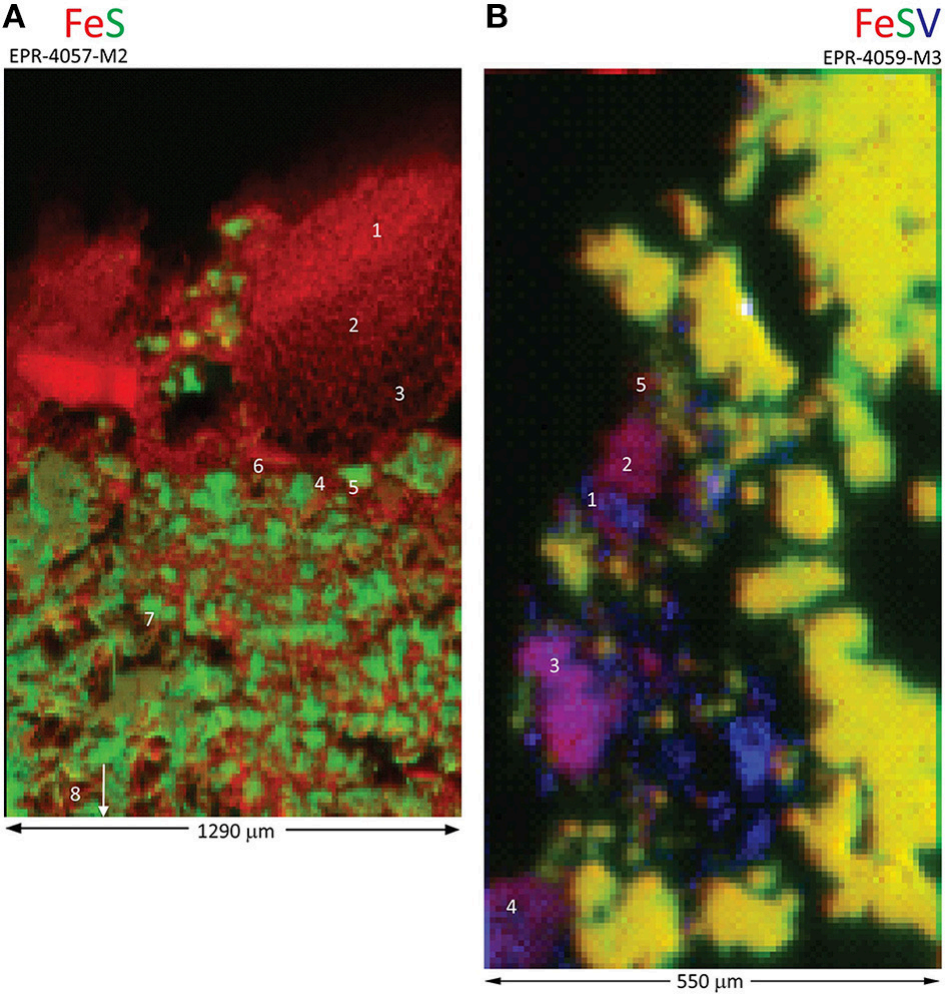
**Distribution of Fe, S, and V in massive sulfide deposits near Bio9 Vent**. X-ray fluorescence maps for **(A)** EPR-4057-M2, and **(B)** red-green-blue (Fe-S-V) distribution for EPR-4059-M3. White numbers indicate locations for Fe X-ray absorption near edge structure (XANES) (Table S3) or extended X-ray absorption fine structure (EXAFS) (Table S4) data collection.

Our sample set includes two massive sulfide deposits in the vicinity of Bio9 Vent (EPR-4057-M2 and EPR-4059-M3; Figures S1C,D). Both deposits exhibit Fe-rich and S-depleted zones at the seawater exposed surfaces with low (or undetectable) Si (Figure 3). The Fe XANES data for EPR-4057-M2 are dominated by the Fe(III) oxyhydroxide goethite with lesser contributions of a biogenic-like Fe oxyhydroxide signature (Table S3). The goethite phase assignment is supported by Fe EXAFS (Table S4) and micro-probe X-ray diffraction (Figure S7). One Fe XANES location is consistent with the Fe(III) oxyhydroxide lepidocrocite (spot 7). Where Fe XANES and EXAFS observations overlap for this sample, the observations are in agreement (Table S4). The Fe XANES data for the seawater exposed portion of massive sulfide EPR-4059-M3 reveal heterogeneous Fe-bearing mineralogy: consistent with 2-line ferrihydrite, goethite, akaganeite, and secondary phyllosilicates (“clay” minerals; Table S3). Despite the complexity in Fe-bearing phases, the Fe-rich and S-depleted phases are predominantly Fe(III).

**Figure 4:**
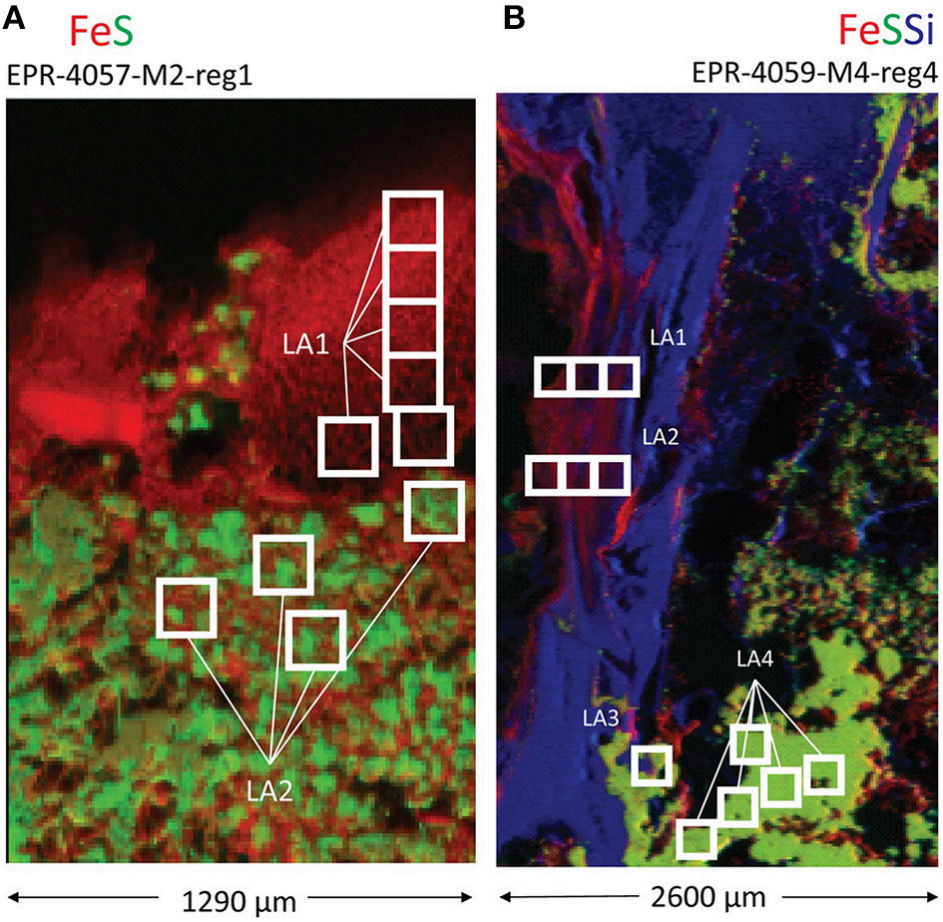
**Locations of laser ablation (LA) sampling for (A) a massive sulfide sample (EPR-4057-M2-reg1) and (B) an extinct, off-axis chimney sample (EPR-4059-M4-reg4)**. X-ray fluorescence (XRF) maps are annotated with white boxes indicating laser ablation areas corresponding to isotopic data in Tables S6, S7.

Light micrographs and X-ray fluorescence maps of region 1 of sample EPR-4057-M2 (Figure S8; Figure 4A) show a relatively sharp contact between the external layer (i.e., alteration crust composed essentially of Fe oxyhydroxides) and the pyrite-rich interior. Disseminated Fe oxyhydroxides also occur as coatings and void filling between pyrite grains suggesting partial pyrite alteration. In this region, average δ^57^Fe values by laser ablation analysis for Fe oxyhydroxides in the alteration layer and pyrite are –1.93 ± 0.20‰ (2 sd, *n* = 6) and –1.69 ± 0.22‰ (2 sd, *n* = 4), respectively. In other regions of the same sample similar average δ^57^Fe values for Fe oxyhydroxides (–1.64 ± 0.32‰) and pyrite (–2.37 ± 0.40 and –2.12 ± 0.60‰) are obtained (Table S6). Average δ^57^Fe values for pyrite are consistent with bulk values determined at –2.58 ± 0.09‰ (Rouxel et al., 2008a). However, differences in δ^57^Fe values from microdrilling another region of the sample are observed (region 3): δ^57^Fe values ranging from –0.25 to –0.44‰ for Fe oxyhydroxides and –1.09‰ for pyrite (Table S6). These results suggest significant isotope heterogeneity of pyrite in this sample and show that Fe oxhydroxides may have either similar or slightly heavier δ^57^Fe values relative to pyrite; which is in contrast with results obtained for sample EPR-4053-M3.

### Inactive off-axis chimney

**Figure 5:**
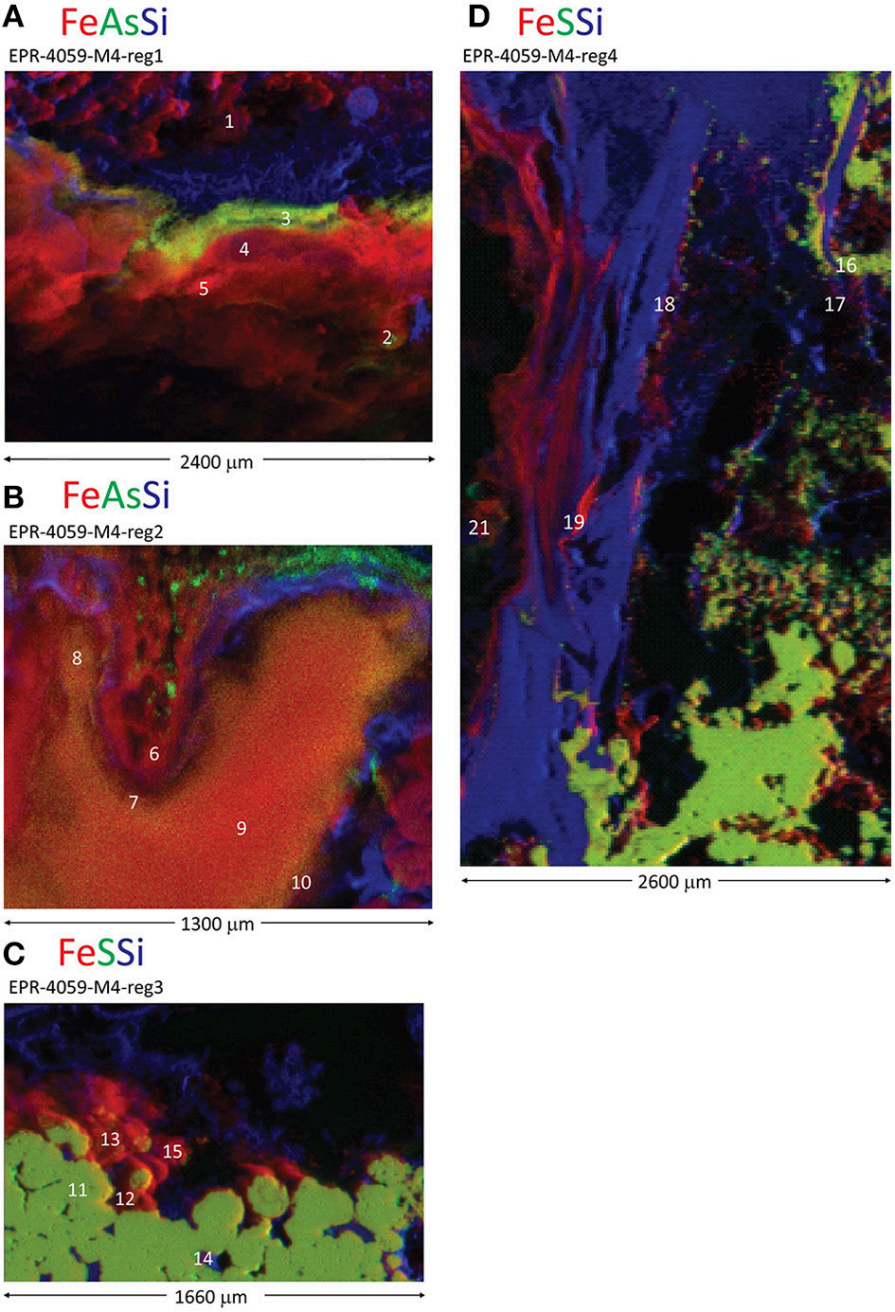
**Distribution of Fe, S, As, and Si in an inactive, off-axis chimney from the East Pacific Rise**. Red-green-blue (Fe-S-Si or Fe-As-Si) tricolor X-ray fluorescence maps for four different regions of the sample: **(A)** EPR-4059-M4-reg1, **(B)** EPR-4059-M4-reg2, **(C)** EPR-4059-M4-reg3, and **(D)** EPR-4059-M4-reg4. White numbers indicate locations for Fe X-ray absorption near edge structure (XANES; Table S3) or extended X-ray absorption fine structure (EXAFS; Table S4) data collection.

**Figure 6:**
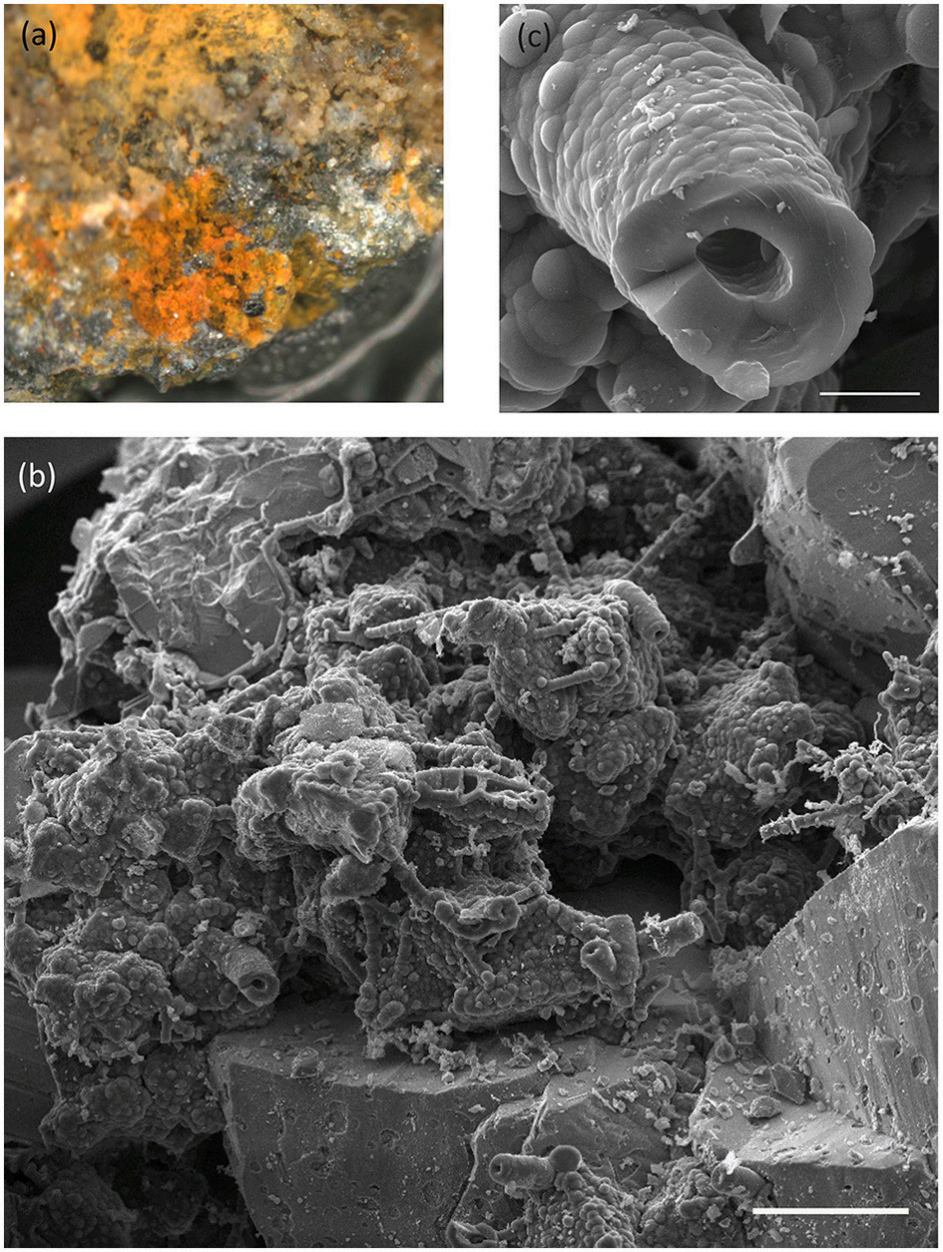
**Images of seawater exposed surface of East Pacific Rise off-axis, extinct chimney sample (EPR-4059-M4). (A)** Binocular light microscope image of sample showing fine-grained orange and yellow alteration products. Field of view is ~0.5 cm. **(B)** Representative scanning electron microscope (SEM) image highlighting the abundance of biological materials. Scale bar is 100 μm. **(C)** A higher magnification image of a stalk-type structure prevalent in these materials. Scale bar is 10 μm.

An extinct, off-axis chimney structure (EPR-4059-M4) located 300 m east of the spreading center is part of our study (Figure S1E). Four regions of one grab sample show (Figures 4B, 5) that the sample is characterized by a complex interweave of Fe(III)- and Si-rich layers. The seawater-exposed portion of the sample had bright orange and yellow precipitates (Figure 6A) composed of biological material (Figures 6B,C).

**Figure 7:**
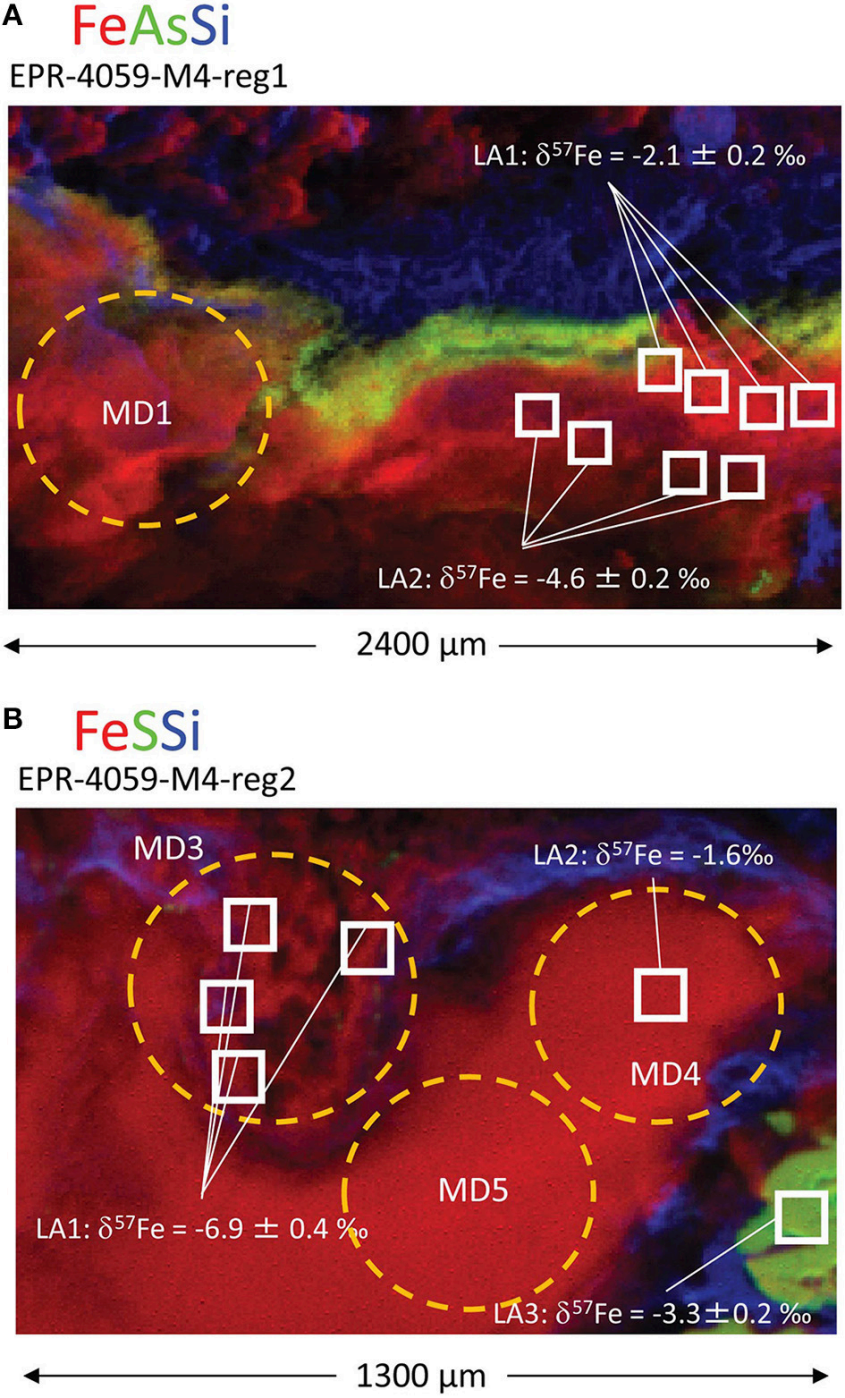
**Locations of laser ablation (LA) and micro-drilling (MD) samples for two regions within the extinct, off-axis chimney: (A) EPR-4059-M4-reg1 and (B) EPR-4059-M4-reg2**. Both laser ablation and micro-drilling produced samples for isotopic analysis (Table S7).

In region 1, the sulfides (pyrite) are overlain by a Si-rich layer, then an As-rich layer, and finally a Fe(III)-rich layer exposed to seawater(Figure 5A, spot 1; Table S3). The Fe XANES data indicate that the Fe(III) layer in region 1 was 70–100 mol% biogenic-like Fe oxyhydroxide with varying contributions from goethite and phyllosilicate (Figure 5A; spot5; Table S3). Laser ablation analyses of pyrite yield average δ^57^Fe values of –2.9 ± 0.7‰ (2 sd, *n* = 7; Table S7; Figure 7A), which is within the range of δ^57^Fe values for the bulk sample (δ^57^Fe = –2.33 and –2.94‰) (Rouxel et al., 2008a). Iron isotope analysis of 3 areas enriched in Fe oxyhydroxides show contrasting Fe isotope values: (i) the external layer enriched in As has Fe isotope values similar to pyrite, with δ^57^Fe = –2.1 ± 0.5‰ (2 sd, *n* = 4); (ii) the internal layer between pyrite and the external As-Fe-Si external crust shows very light Fe isotope values, with δ^57^Fe = −4.6 ± 0.3‰ (2 sd, *n* = 4) which is even lighter than pyrite values; and (iii) average Fe oxyhydroxide layer (i.e., sampled by microdrilling) adjacent to the two others has a heavier value, δ^57^Fe = –0.95 ± 0.04‰.

Region 2 is internal to the grab sample and has Fe(III)-, As-, and S-rich layers (Figure 5B). The Fe oxyhydroxides in this region do not occur as a well-defined external crust along the outside layer of the chimney wall, but rather as filling material in a chimney cavity probably exposed to ambient seawater. The Fe XANES for the Fe(III)-rich materials are consistent with biogenic-like Fe oxyhydroxide, 2-line ferrihydrite, and goethite (Table S3). For region 2 spot 21, the best one component fit to the Fe EXAFS data is biogenic-like Fe oxyhydroxide (Table S4), and the best two component fit is biogenic-like Fe oxyhydroxide plus phyllosilicate. For the EXAFS LCF, the one component fit is chosen based on the *a priori* requirement that additional fit components require a 10% or greater improvement in the normalized sum square (NSS) parameter. The EXAFS results are overall consistent with the Fe XANES fitting. This region shows the lightest Fe isotope values obtained so far in natural samples with δ^57^Fe values obtained by laser ablation ranging from −6.9 ± 0.7‰ (2 sd, *n* = 4) to –1.6 ± 0.3‰ while values from microdrilling ranging from −4.73 to −1.83‰ (Figure 7B; Table S7). This demonstrates a great heterogeneity of Fe isotopes values, characterized by δ^57^Fe values both lighter and heavier than pyrite values from the adjacent area (δ^57^Fe = –2.54‰ and –3.32 ± 0.4‰, microdrilling and laser ablation, respectively).

Region 3 has a thin layer of Fe(III) overlaying sulfides, with the seawater-exposed edge covered by a thin Si-rich layer (Figure 5C). The Fe XANES from the sulfide-Fe(III) transition indicate a variety of Fe(III)-bearing phases including biogenic-like Fe oxyhydroxide, goethite, and phyllosilicate (Table S3). No Fe isotope analysis was obtained from this region.

In region 4, sulfides (pyrite) are overlain by Si- and then Fe(III)-rich layers (Figure 5D, spot 16; Table S3). The Fe XANES indicate that the Fe(III)-bearing phases with spectral signatures consistent with 2-line ferrihydrite, goethite, akaganeite, and phyllosilicate (Table S3). Iron isotope values for pyrite obtained by laser ablation are relatively homogenous, with δ^57^Fe = –3.25 ± 0.7‰ (2 sd, *n* = 12), which is similar within uncertainties to the lower values obtained for microdrilled pyrite (δ^57^Fe = −3.28‰) and bulk pyrite using (δ^57^Fe = −2.33 and −2.94‰; Table S7; Figure 4B) (Rouxel et al., 2008a). Iron oxyhydroxides overlying the Si-rich layer show larger spread of δ^57^Fe values, from −2.71 ± 0.12‰ (microdrilling) to −4.1 ± 0.8‰ (*n* = 3; laser ablation).

### Diversity of iron-bearing phases and co-located elements

**Figure 8:**
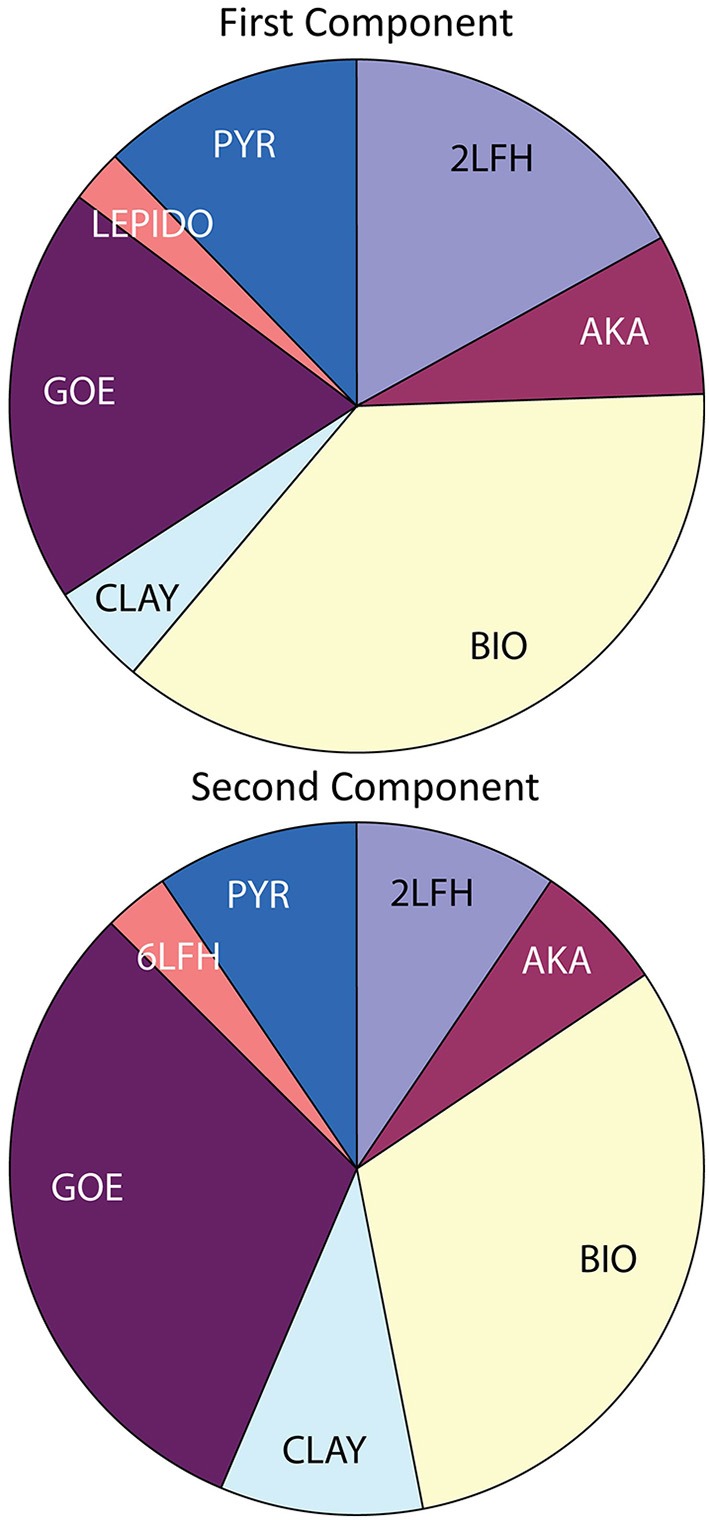
**Summary of linear combination fitting (LCF) results for Fe XANES spectra**. Acronyms described in Table S2 and full data set displayed in Table S3.

A summary of the linear least-squares fitting of spectra to combinations of reference spectra (LCF) is provided in Table S3. Principal component analysis (PCA) for the 43 Fe XANES spectra is consistent with eight different Fe species; as indicated by the minimum of the *IND* parameter (Manceau et al., 2002). The LCF results for Fe XANES spectra are in agreement with the PCA assessment of eight different Fe species among the 1st and 2nd fit components (Table S3; Figure 8). For 78% of the spectra, a second component in the fit is justified by a >10% improvement of the NSS parameter. For 32% of the spectra, a 3rd component is justified. These results indicate that in most cases, even at the 10-micron scale, a mixture of Fe-bearing minerals is present. The spectral components supported by PCA, target transformation analysis (TTA), and LCF are: pyrite (PYR); biogenic Fe oxyhydroxide (BIO); lepidocrocite (LEPIDO); 6-line ferrihydrite (6LFH); 2-line ferrihydrite (2LFH); nontronite (NONT); smectite (CLAY-S); goethite (GOE); and akaganeite (AKA) (Table S2). Note that Fe-bearing phyllosilicates nontronite and smectite components are binned as a single “clay” category (Figure 8). Three additional minor Fe-species (never exceeding 13 mole%) are supported by LCF as 3rd components: chromite (CHROM); richterite (RICHT); and pervoskite (PEROV). These fit results indicate that some additional Fe(II) is present in the sample, but are not interpreted as firm evidence for the specific mineral form.

### Comparison of iron XANES and EXAFS data

**Figure 9:**
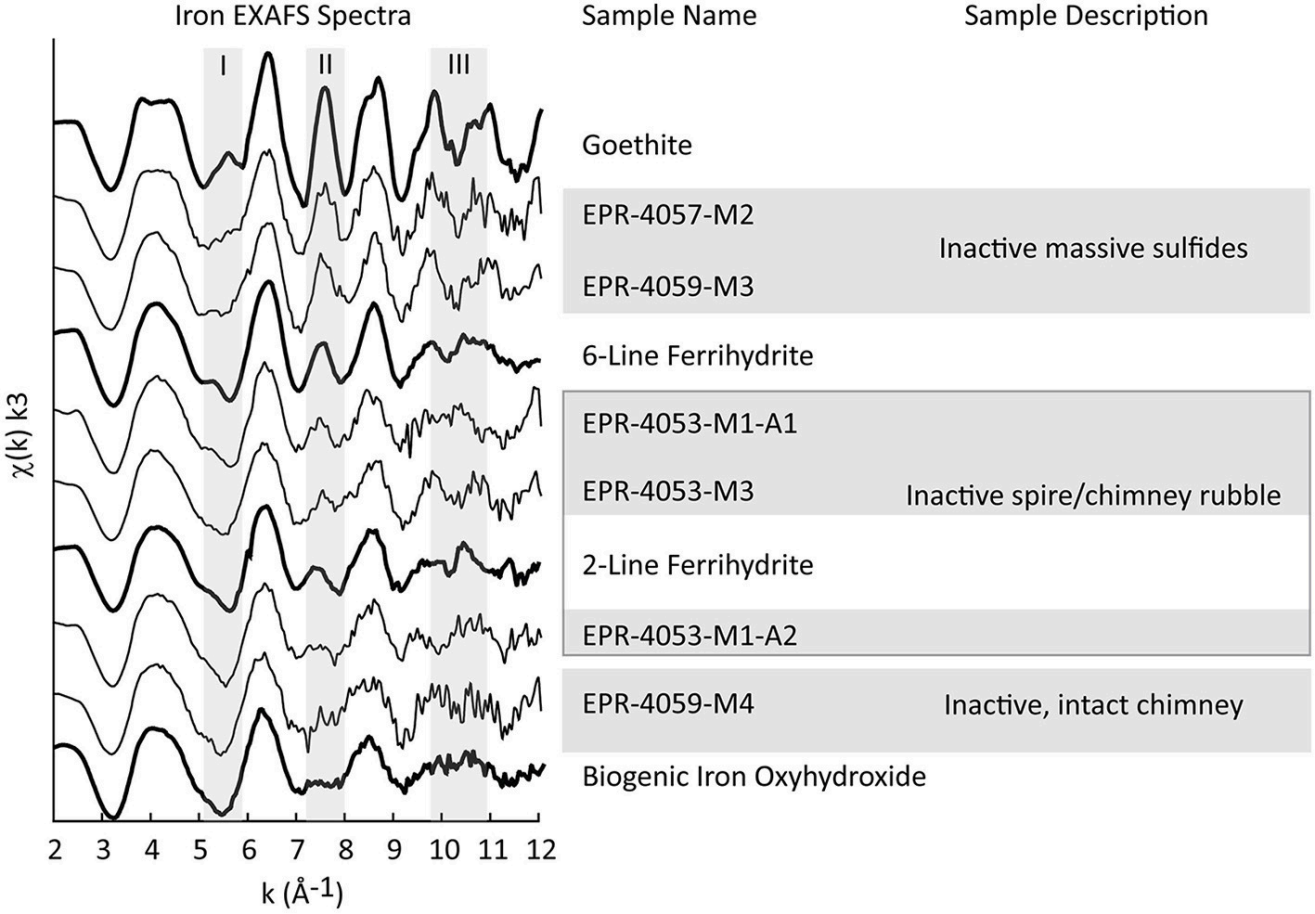
**Iron EXAFS spectra for EPR samples (fine lines) are plotted with spectra from Fe-bearing reference materials (bold lines)**. The location of Fe EXAFS data collection is recorded in Table S4 with asterisks and annotated in Figures 1, 3, 5.

A full continuum of Fe oxyhydroxide phases, supported by Fe EXAFS data, are observed for the EPR sulfide deposits, from BIO-like phases lacking long-range structural order to well-crystallized goethite (Figure 9). Like the Fe XANES, Fe EXAFS spectra are subjected to LCF with reference spectra. Shell-by-shell fitting of the EXAFS data was not attempted due to the relatively short k-space range of the useable data (ca. 11 Å^−1^). The results of the fitting are summarized in Table S4, and the LCFs are plotted with the EXAFS data in Figure S9. The fits to the EXAFS data agree well with XANES fits for the same locations on the samples. For example, the Fe EXAFS data from sample EPR-4057-M2 is best fit by 67% goethite (GOE) and 39% biogenic Fe oxyhydroxide (BIO) references. In this sample location, microprobe XRD was used to verify that goethite was present (Figure S7); this is an important methodological confirmation that the goethite reference spectrum is representative of goethite in the sample. The corresponding fit to the much shorter Fe XANES data region yields 62% goethite and 34% BIO. The comparisons of XANES and EXAFS data also show that spectroscopically similar phases can be difficult to distinguish using LCF. For example, the Fe EXAFS data from sample EPR-4053-M1-A2 is best fit by 55% BIO and 34% 6-line ferrihydrite (6LFH), while the corresponding Fe XANES data fit yields 47% BIO and 52% 2-line ferrihydrite (2LFH).

### Comparison of iron isotope compositions

**Figure 10:**
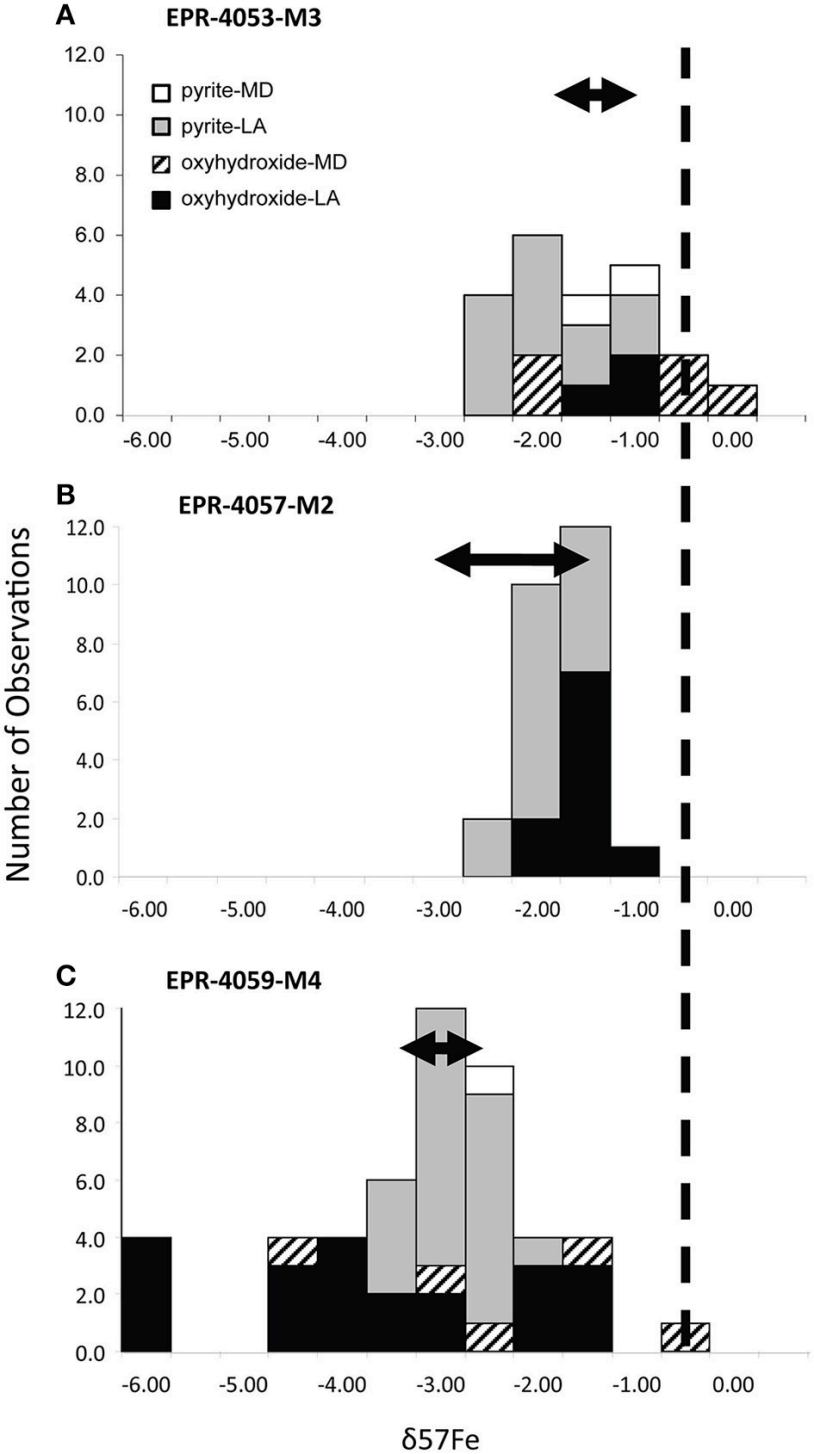
**Summary of δ^**57**^Fe isotope values for (A) EPR-4053-M3 (K Vent rubble), (B) EPR-4057-M2 (Bio9 Area massive sulfide), and (C) EPR-4059-M4 (extinct, off-axis chimney)**. Micro-drilling (MD), laser ablation (LA), pyrite, and oxyhydroxides sample characteristics are indicated in the legend.

The EPR chimney samples that exhibit complex interweaved layers of Fe(III), Si, and sulfide have a larger range of δ^57^Fe values relative to massive sulfides (e.g., EPR-4057-M2). When Fe oxyhydroxides occur as filling materials within the chimney wall or as outside layer crusts in association with Si-rich material, enrichments in both heavy and light Fe isotopes relative to pyrite are possible. The range of values δ^57^Fe is up to 6‰ which enlarges not only the range of Fe isotopes measured in hydrothermal vent environments (Rouxel et al., 2008a) but also in marine sediments (Severmann et al., 2006; Johnson et al., 2008; Rouxel et al., 2008b). In most cases, Fe-oxyhydroxides are enriched in light Fe isotopes relative to vent fluid sources (Figure 10).

## Discussion

### Iron isotope systematics

Significant variability of Fe isotope composition in (oxyhydr)oxide precipitates is expected when Fe(II) is partially oxidized in conditions with slow rates of oxidation. Microbiological experiments have shown that Fe isotope fractionations are produced during dissimilatory Fe(III) reduction (Beard et al., 2003b; Icopini et al., 2004; Johnson and Beard, 2005) and anaerobic photosynthetic Fe(II) oxidation (Croal et al., 2004; Swanner et al., 2015). Iron isotopes can also be fractionated by abiotic Fe(II) oxidation, precipitation of ferric hydroxides (Bullen et al., 2001; Beard et al., 2010), and by sorption of aqueous Fe(II) onto Fe oxyhydroxides (Icopini et al., 2004; Wu et al., 2012). The largest equilibrium isotope fractionations (~4.5‰ in ^57^Fe/^54^Fe ratios) observed and theoretically calculated are for coexisting Fe(III) and Fe(II) aqueous species (Welch et al., 2003; Anbar et al., 2005). Hence, in view of the variety of fractionating processes during Fe redox changes, it is not surprising that marked variations in Fe-isotope composition have been observed in Fe-rich marine environments (Severmann et al., 2006; Rouxel et al., 2008b). Large variations are also reported in banded iron formations (Johnson et al., 2003; Rouxel et al., 2005; Dauphas and Rouxel, 2006; Planavsky et al., 2012), hydrothermal fluids and precipitates (Sharma et al., 2001; Beard et al., 2003a; Rouxel et al., 2003, 2004), and ancient seafloor hydrothermal Fe-Si deposits (Planavsky et al., 2012; Moeller et al., 2014). Iron (oxyhydr)oxide particles within near-vent (buoyant) hydrothermal plumes at the Rainbow hydrothermal field (Mid Atlantic Ridge) have variable δ^57^Fe values (0.15 to 1.65‰) relative to the original vent fluid, consistent with fractionation during partial oxidation of Fe(II)*aq* to Fe(III)*aq* in seawater (Severmann et al., 2004). In contrast, Fe (oxyhydr)oxide-rich sediments precipitated from non-buoyant hydrothermal plumes have δ^57^Fe values that are indistinguishable from that of high-temperature hydrothermal fluids (Severmann et al., 2004). In a more recent study, low-temperature Fe (oxyhydr)oxide deposits from the Jan Mayen hydrothermal vent fields Norwegian-Greenland Sea yielded very light δ^57^Fe values down to −2.8‰ (Moeller et al., 2014). These values are indistinguishable from low-temperature hydrothermal fluids from which they precipitated, suggesting that hydrothermal vent fluid underwent significant partial Fe(II) oxidation below seafloor leading to isotopically light Fe values for Fe(II), as previously proposed (Rouxel et al., 2003).

In our study, δ^57^Fe values for Fe oxyhydroxides of the chimney wall are both heavier and lighter than the coeval pyrite or hydrothermal fluids. We propose that a reservoir effect is created during partial Fe(II) oxidation and can explain the diversity of values: (1) heavier values are consistent with small extent of Fe(II) oxidation; (2) lighter values are consistent with a greater extent of Fe(II) oxidation; and (3) lightest values are consistent with loss of Fe oxyhydroxides along a flow path (open system Fe oxidation) (Dauphas and Rouxel, 2006; Moeller et al., 2014). Extremely light Fe isotope values can be attained through Rayleigh-type fractionation and could explain the δ^57^Fe values, as low as −7‰, we observe for Fe oxyhydroxides in sample EPR-4059-M4 (Figure 10C). Such partial Fe(II) oxidation probably required micro-aerobic conditions in which the rate of Fe(II) oxidation is slow enough to generate high variability of δ^57^Fe values. Considering that such conditions are also favorable for the growth of Fe-oxidizing microorganisms (Druschel et al., 2008), the involvement of microorganisms in the Fe transformation pathways is likely. However, biotic Fe oxidation is not demonstrated in the present case because abiotic Fe oxidation can also produce these Fe isotope fractionation factors.

### Iron transformation pathways in sulfide mineral deposits

#### Summary of transformation pathways

The main goal of this study is to describe the Fe transformation pathways in natural sulfide mineral deposits at the seafloor, and examine the transformation products for indications or markers of biological activity. The hydrothermally inactive sulfide deposits of the EPR 9–10°N are known to host microbial communities with the genetic potential to alter Fe- and S-bearing minerals through redox reactions (Sylvan et al., 2012; Toner et al., 2013). Our investigation of the EPR sulfide deposits indicates that the alteration products are generated by at least four different pathways. Each pathway can be *complete and quantitative*, leading to an isotopic signature similar to the source Fe, or *partial*, leading to isotopic fractionation:

(Pathway 1) direct precipitation of Fe(II)*aq*, to form primary sulfide minerals, from hydrothermal fluids in zones of mixing between vent fluids and seawater;
(Pathway 2) precipitation of Fe(II)*aq*, to form sulfide minerals, from Fe(III) reduction in zones of mixing between vent fluids and seawater;
(Pathway 3) direct oxidation of Fe(II)*aq* from hydrothermal fluids, to form Fe(III) precipitates, in zones of mixing between vent fluids and seawater; and
(Pathway 4) oxidative alteration of pre-existing sulfide minerals to form Fe(III) precipitates.

#### Pathways created by vent fluid-seawater mixing

In several sulfide deposits at the EPR, we observe layered zones of Fe(III)-, Si-, and sulfide-rich precipitates that are consistent with cooling vent fluids and low redox potential (e.g., Figures 1A,D,C, 5). The precipitation of pyrite in the exterior chimney wall is probably a late-stage phenomenon reflecting a different pathway of mineral formation than the coarse-grained to euhedral pyrite composing the chimney wall. In many cases, we observe colloform and spherulitic pyrite textures on the chimney exterior in association with amorphous silica and often lining former worm tubes. Fine-grained to colloform pyrite with minor sphalerite may occur along fossil worm tubes (formed probably by *Alvinella*) (Little et al., 2007; Rouxel et al., 2008a). As discussed by Xu and Scott, 2005, spherulitic and colloform textures of pyrite reflect rapid crystallization (i.e., disequilibrium) with cooling caused by mixing between hot vent fluid and cold ambient seawater. Overall, for our samples, the texture and morphology of these pyrite-Si-oxyhydroxide zones is highly variable; we speculate that this is caused primarily by the wide range of fluid flow and redox regimes created in actively venting chimneys. For many of our samples, pyrite lined fossil worm tubes exhibit δ^34^S and δ^57^Fe values (4.3 to 3.0 and −1.8 to −1.2‰, respectively) typical of bulk pyrite from the chimney wall (Rouxel et al., 2008b), which is consistent with direct precipitation from hydrothermal fluids (Pathway 1).

In addition to Pathway 1, the presence of pyrite within layered zones of Fe(III)-, Si-, and sulfide-rich precipitates could be caused by reductive processes in the mixing zone (Pathway 2). For example, pyrite forming at low–temperature and in an open system in contact with sulfate-rich seawater creates the potential for microbial sulfate and Fe(III) reduction. This possibility has been proposed from studies of microbial diversity in hydrothermal chimneys (Callac et al., 2015), as well as metabolic energetic calculations (Amend et al., 2011). Sulfur isotope studies show the contribution of microbial sulfate reduction and sulfide formation at mid-oceanic ridges (Peters et al., 2010); however, a robust S isotope biosignature in hydrothermal chimney environments has not been demonstrated. Our results provide strong evidence for secondary and late-stage pyrite formation along the outside wall of the chimneys due to vent fluid-seawater mixing. This interpretation, based on isotope data, Fe speciation, and mineral textures, is also consistent with the observed enrichment in silica. In general, silica precipitation in seafloor hydrothermal chimneys requires some degree of conductive cooling due to the solubility of amorphous Si in simple mixing between hydrothermal fluids and seawater (Hannington et al., 1995).

Iron oxyhydroxides may also form in low-oxygen environments during hydrothermal-fluid mixing, although the expected slow rate of Fe oxidation in dilute hydrothermal fluids may limit the accumulation of Fe oxyhydroxides in the absence of microbial mediation. Localized Si and As enrichments within the Fe oxyhydroxides is common for our samples (Figures 1A,C,D, 5A–D). In many cases, the layered Fe(III)-, Si-, and sulfide-rich zones exhibit δ^57^Fe values that are consistently lighter than hydrothermal vent fluids (Figure 10). This observation cannot be explained by either partial or quantitative Fe(II) oxidation because the resulting Fe(III) should be enriched in heavy Fe isotopes relative to Fe(II), regardless of the mechanism and extent of Fe(II) oxidation. Therefore, it is likely that these Fe oxyhydroxides formed through a combination of quantitative oxidation of vent fluid Fe(II) (Pathway 3) and alteration (i.e., oxidation) of pyrite characterized by much lighter δ^57^Fe values than vent fluids (Pathway 4).

In sample EPR-4053-M3-reg1 (Figure 2), δ^57^Fe values are consistent with either partial oxidation of pyrite (*partial* Pathway 4) or direct and quantitative oxidation of late-stage vent fluid Fe(II) (Pathway 3). The δ^57^Fe values of the Fe oxyhydroxides range from −0.32 to −1.59‰ which is heavier than associated pyrite by up to 2.3‰ but also within the range expected for hydrothermal vent fluids, especially low-temperature or late-stage vent fluids (Rouxel et al., 2008a; Moeller et al., 2014). In contrast, sample EPR-4059-M4-reg2 (Figure 7B) exhibits very light Fe isotope values for the Fe oxyhydroxides, which are up to 4.7‰ lighter than associated pyrite. These data cannot be explained by simple mixing between Fe(III) derived from local sulfide (pyrite) oxidation and Fe(II) from vent fluid. During partial Fe(II) oxidation, the remaining Fe(II) in solution is expected to become isotopically lighter due to the precipitation of isotopically heavy Fe-oxyhydroxide (*partial* Pathway 3). This mechanism, often referred to as a reservoir effect, has been shown to lead to isotopically light Fe(II) in sediment porewater (δ^57^Fe down to −7.38‰) (Rouxel et al., 2008a) and likely occurs within cavities of hydrothermal chimneys. The fact that isotopically light Fe oxyhydroxides occur in association with significant Si enrichment (e.g., EPR-4059-M4-reg4; Figure 4B) and filling cavities of the chimney wall (EPR-4059-M4-reg2; Figure 7B) is consistent with this assumption. In this case, Fe(II) released during pyrite alteration (Pathway 4) or late-stage hydrothermal fluid undergoing partial oxidation in the chimney wall (*partial* Pathway 3), leads to the formation of isotopically light Fe(II) diffusing out of the chimney wall and ultimately precipitating as Fe oxyhydroxide.

#### Pathways created by complete and direct oxidation

In contrast to the Fe transformation pathways discussed in section Pathways Created by Vent Fluid-Seawater Mixing, the two massive sulfide deposits exhibit a simpler set of Fe reactions. For the EPR-4057-M2-reg1, the δ^57^Fe values are very close to Fe in unaltered pyrite (−1.93 ± 0.20 vs. −1.69 ± 0.11‰), and are consistent with direct and complete oxidation of pyrite-Fe to goethite-Fe (Pathway 4; Figure 4A). While the incipient Fe oxyhydroxide phase is not known in this case, a short-term (2 months) chimney incubation study at the Juan de Fuca Ridge indicates the possibility of a biogenic-like Fe oxyhydroxide incipient phase (Toner et al., 2009b). The fact that the Fe oxyhydroxide is primarily goethite, with no co-located Si or As, is suggestive of transformation of the initial precipitation products to the stable goethite phase over time in ambient deep ocean waters.

## Conclusions

One of the main objectives of the present study is to determine whether alteration materials associated with seafloor sulfide deposits possess mineralogical or stable isotope biosignatures. In essence, we want to know the reactants, mechanisms, and products for reactions involving Fe. In light of the microbial community data (Sylvan et al., 2012; Toner et al., 2013), textural complexity noted in hand specimens (Rouxel et al., 2008a), and spatial scale of mineralogical variability in petrographic sections (this work), we chose a spatially resolved spectroscopic and isotopic approach to the study of the EPR sulfides. The spectroscopic approach provides the reaction products (and in some cases reactants) through measurement of the chemical form of Fe, as well as the identity of co-located elements: while the isotopic approach provides the source-Fe reactant(s) and the extent of the reaction. The application of these complementary tools at the 10–50 micron spatial scale certainly must have averaged over finer submicron geochemical information. However, the spatial scale of investigation did allow us to describe the diversity of mineral forms and isotopic signatures intrinsic to these sulfides, and demonstrates the synergy of spectroscopic and isotopic approaches.

Culture-independent microbiology results show that microbial communities of the EPR change when chimney structures become hydrothermally extinct (Sylvan et al., 2012). Once extinct, the EPR sulfide deposits host bacterial communities similar in composition and structure to mid-ocean ridge sulfides from far distant sites, such as the Indian Ocean (Toner et al., 2013). The presence of bacterial groups known for Fe and S cycling in EPR sulfide samples (e.g., β-Proteobacteria *Gallionella* sp. and ε-Proteobacteria *Sulfurimonas* sp.) indicates that microorganisms should be able to mediate the alteration of sulfide mineral substrates. As a result of S and/or Fe oxidation processes, Fe(III) reaction products are expected, and Fe(III)-bearing phases are abundant in the EPR sulfide deposits examined in this study. Overall, these findings provide evidence to support the idea that inactive sulfide deposits are more than passive surfaces for microbial attachment. However, only the genetic potential, not the metabolic capabilities, of microbial community members are measured by the sequencing approaches used to-date for this sample set.

While our analysis of Fe mineralogy and isotope values do not support or refute a unique biological role in Fe(III) oxyhydroxide precipitation during sulfide alteration, our findings do reveal complex reaction pathways of Fe—precipitation/dissolution and oxidation/reduction—in seafloor sulfide deposits. The variety of Fe transformation pathways we observe is consistent with the development of micro-environments within the sulfide deposits. By defining four pathways of Fe transformation, we can propose several micro-environments. We observe zones of mixing between vent fluids and seawater with both reducing (Pathways 1 and 2) and oxidizing conditions (Pathways 2 and 4). These micro-environments are consistent with the diverse genetic potential, and correspondingly wide range of potential metabolisms, observed in organisms cultured from sulfide deposits. The reducing-to-oxidizing range of mixing zones, in particular, could support micro-environments favorable to Fe and S oxidation and reduction within a single chimney deposit.

In contrast to the dynamic multi-step pathways revealed by Fe isotopes, the Fe XAS observations for these samples are consistent with stabilization of poorly ordered Fe(III) oxyhydroxide phases when Si and As are present. At first glance, it is surprising that multi-stage Fe oxidation and precipitation processes would preserve poorly ordered, and presumably, metastable Fe(III) oxyhydroxide phases. However, laboratory experiments have demonstrated that Fe atom replacement within Fe(III) oxyhydroxides, such as goethite, can produce the same mineral structure and particle morphology in the presence of Fe(II)*aq* (Handler et al., 2009). While there are many time-dependent factors that our measurements cannot assess—temperature, flow-rate, time-resolved vent fluid chemistry—the dynamic history supported by the Fe isotopes and static history supported by the mineralogy leads us to conclude that inorganic ligands (As, Si), and possibly biological material (Toner et al., 2009b, 2012), stabilize poorly ordered Fe(III) oxyhydroxide phases in these deposits. In addition, ligands appear to be retained by the deposit throughout a complex set of Fe transformation events and dynamic re-working of the Fe-bearing alteration products. It is possible that this mineral-stabilization outcome is driven by a decrease in permeability of the sulfide deposits as a function of time. Low-flow conditions could lead to the retention of ligands in pore spaces and preservation metastable phases despite continued alteration of the Fe-bearing minerals. The physical and chemical environments created by seafloor sulfide deposits do not appear to provide unique biosignatures. However, they do appear to be promising recorders of micro-environmental conditions present during hydrothermal activity.

## Author contributions

Authors CS, OR, WB, and KE collected the samples, OR analyzed samples for Fe and S isotopes, BT and CS analyzed samples for Fe mineralogy, and BT, OR, CS, and WB prepared the manuscript.

### Conflict of interest statement

The authors declare that the research was conducted in the absence of any commercial or financial relationships that could be construed as a potential conflict of interest.

## Supplementary material

Figure S1 **Seafloor photographs of sampling sites. (A,B)** Location of K Vent samples EPR-4053-M2, EPR-4053-M1-A1, and EPR-4053-M1-A2. **(C,D)** Massive sulfide deposits in the Bio9 Vent area EPR-4057-M2 and EPR-4059-M3, respectively. **(E)** Off axis, extinct chimney sources of sample EPR-4059-M4. Images previously published in various forms in Rouxel et al., 2008a; Sylvan et al., 2012; Toner et al., 2013.

Figure S2 **Shipboard photographs of samples**.

Figure S3 **Shipboard photographs of K Vent inactive spire showing the locations of EPR-4053-M1-A1 and EPR-4053-M1-A2**. Portions of this figure are published in Rouxel et al., 2008b

Figure S4 **Photographs of polished thick sections showing the regions of the sample investigated**.

Figure S5 **Example laser ablation run**.

Figure S6 **Iron isotope values as a function of S:Fe and Si:Fe**.

Figure S7 **(A)** Synchrotron radiation microprobe X-ray diffraction data from spot 2 of EPR-4057-M2. **(B)** Data with goethite reference lines.

Figure S8 **Petrographic microscope images of sample EPR-4057-M2. (A)** Iron oxyhydroxide accumulations at the seawater exposed surface of the massive sulfide deposit. **(B)** Oxidation of pyrrhotite crystals. **(C,D)** Sulfide minerals.

Figure S9 **Summary of Fe EXAFS data (dots) and best linear combination fit (solid lines)**. Goodness of fit parameters (Table S4) and key for reference materials (Table S2) are provided in the main text.

Table S1 **Summary of sample locations and naming conventions**.

Table S2 **Summary of Fe-bearing reference materials used for linear combination fitting (LCF) of X-ray absorption spectra**. The χ^2^, *R*-value, and SPOIL parameters are derived from principal component (PCA) and target transformation analysis (TTA).

Table S3 **Linear combination fit results for Fe X-ray absorption near edge structure (XANES) spectra**. Fit components are listed along with the mol% attributed to each component. The components (reference spectra) contributing to the best fit are described in Table S2. The normalized sum square (NSS) parameter is an indicator of the goodness of the fit. An ^*^ symbol indicates that extended X-ray absorption fine structure (EXAFS) spectra were also collected (Table S4). The “Over Absorption Correction” is defined in the Section Synchrotron Microprobe X-ray Absorption Spectroscopy (XAS) Analysis.

Table S4 **Linear combination fit results for Fe extended X-ray absorption fine structure (EXAFS) spectra**. Fit components are listed along with the mol% attributed to each component. The components (reference spectra) contributing to the best fit are described in Table S2. The chi square parameter (χ^2^) is an indicator of the goodness of the fit.

Table S5 **Iron and S isotope analyses for sample EPR-4053-M3-reg1 (K Vent)**. Laser ablation (LA) and micro-drill (MD) sampling are organized according to the dominant mineralogy at that location (pyrite and oxyhydroxide). Specific locations of sampling are displayed in Figure 2. One standard deviation (σ) is reported.

Table S6 **Iron isotope analyses for sample EPR-4057-M2 (Bio9 Area)**. Laser ablation (LA) and micro-drill (MD) sampling are organized according to the dominant mineralogy at that location (pyrite and oxyhydroxide). Specific locations of sampling are displayed in Figure 4A. One standard deviation (σ) is reported.

Table S7 **Iron isotope analyses for sample EPR-4059-M4 (off-axis chimney)**. Laser ablation (LA) and micro-drill (MD) sampling are organized according to the dominant mineralogy at that location (pyrite and oxyhydroxide). Specific locations of sampling are displayed in Figures 4B, 7A,B. One standard deviation (σ) is reported.
