# Supplementary figures and images for: Iron Transformation Pathways and Redox Micro-Environments in Seafloor Sulfide-Mineral Deposits: Spatially Resolved Fe XAS and δ57/54Fe Observations
Source: Front Microbiol. 2016 May 10;7:648. doi: 10.3389/fmicb.2016.00648 (PMC4862312; doi:10.3389/fmicb.2016.00648)

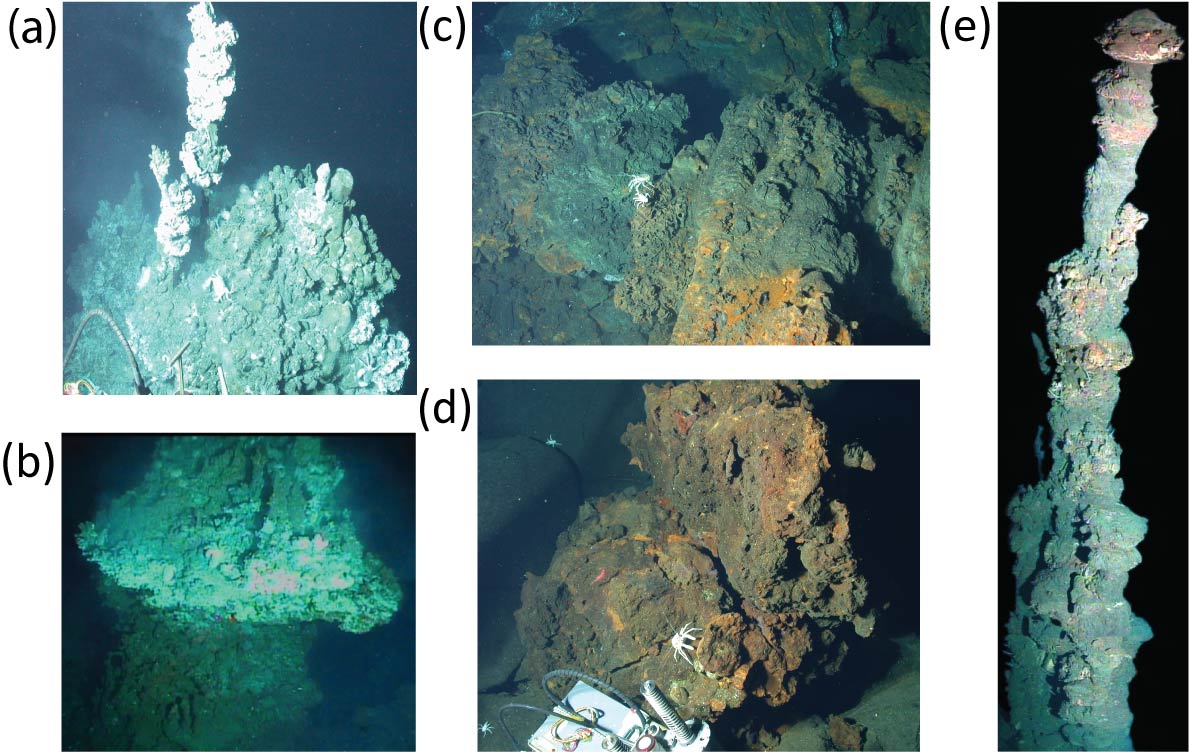

Supplement: Figure S1 — Seafloor photographs of sampling sites. (A,B) Location of K Vent samples EPR-4053-M2, EPR-4053-M1-A1, and EPR-4053-M1-A2. (C,D) Massive sulfide deposits in the Bio9 Vent area EPR-4057-M2 and EPR-4059-M3, respectively. (E) Off axis, extinct chimney sources of sample EPR-4059-M4. Images previously published in various forms in Rouxel et al., 2008a; Sylvan et al., 2012; Toner et al., 2013. [file Image1.JPEG]

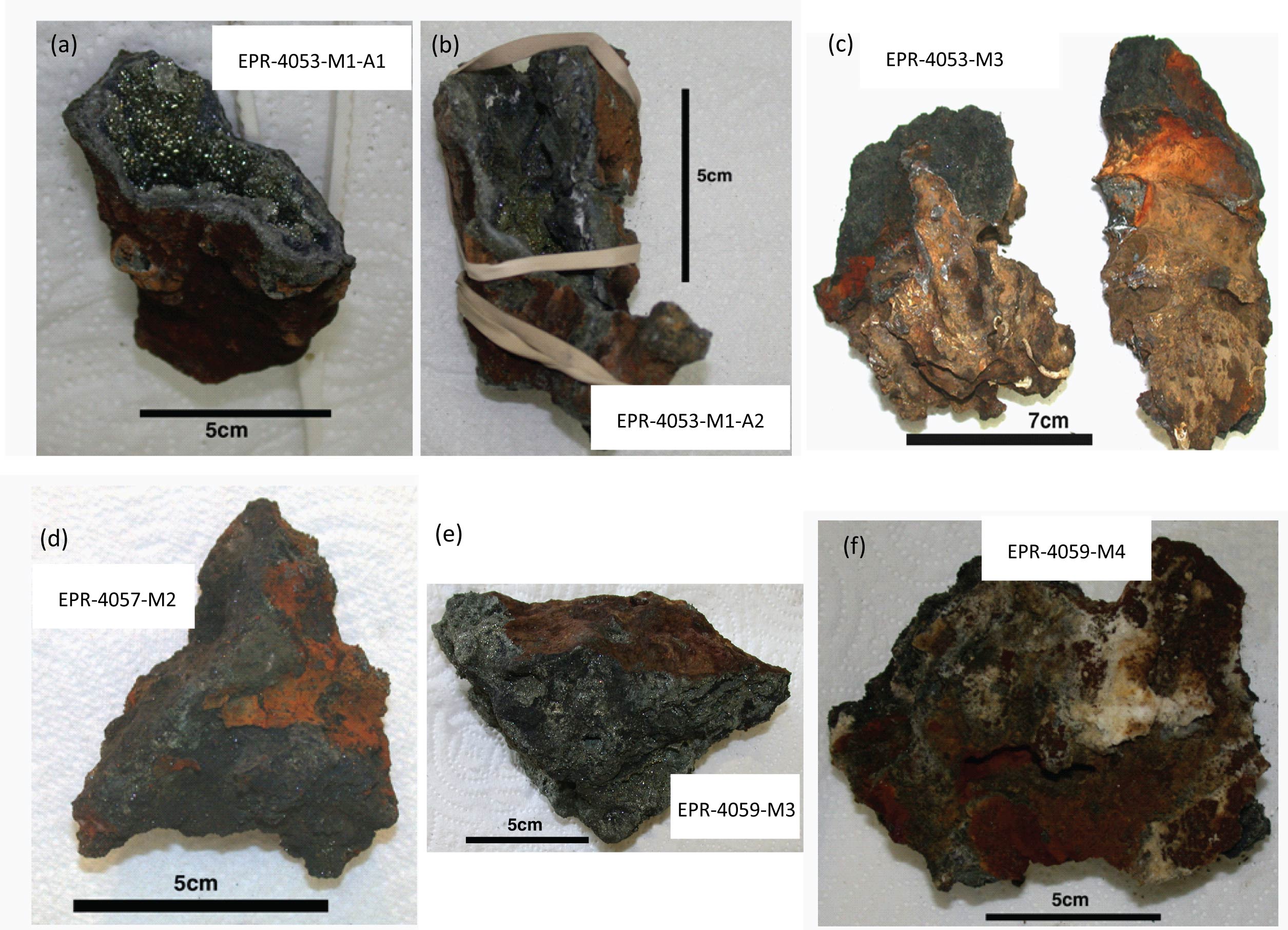

Supplement: Figure S2 — Shipboard photographs of samples. [file Image2.JPEG]

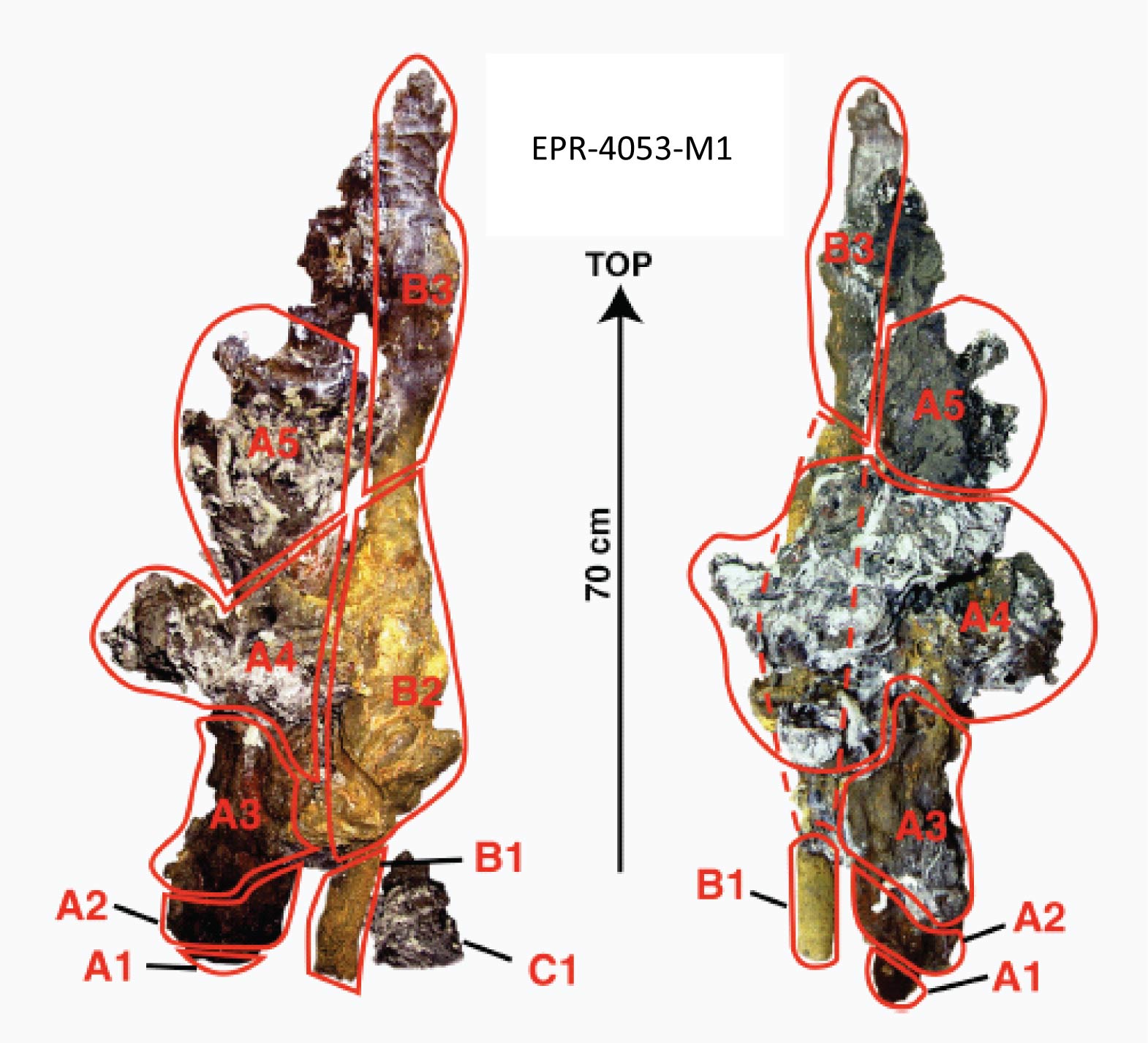

Supplement: Figure S3 — Shipboard photographs of K Vent inactive spire showing the locations of EPR-4053-M1-A1 and EPR-4053-M1-A2. Portions of this figure are published in Rouxel et al., 2008b [file Image3.JPEG]

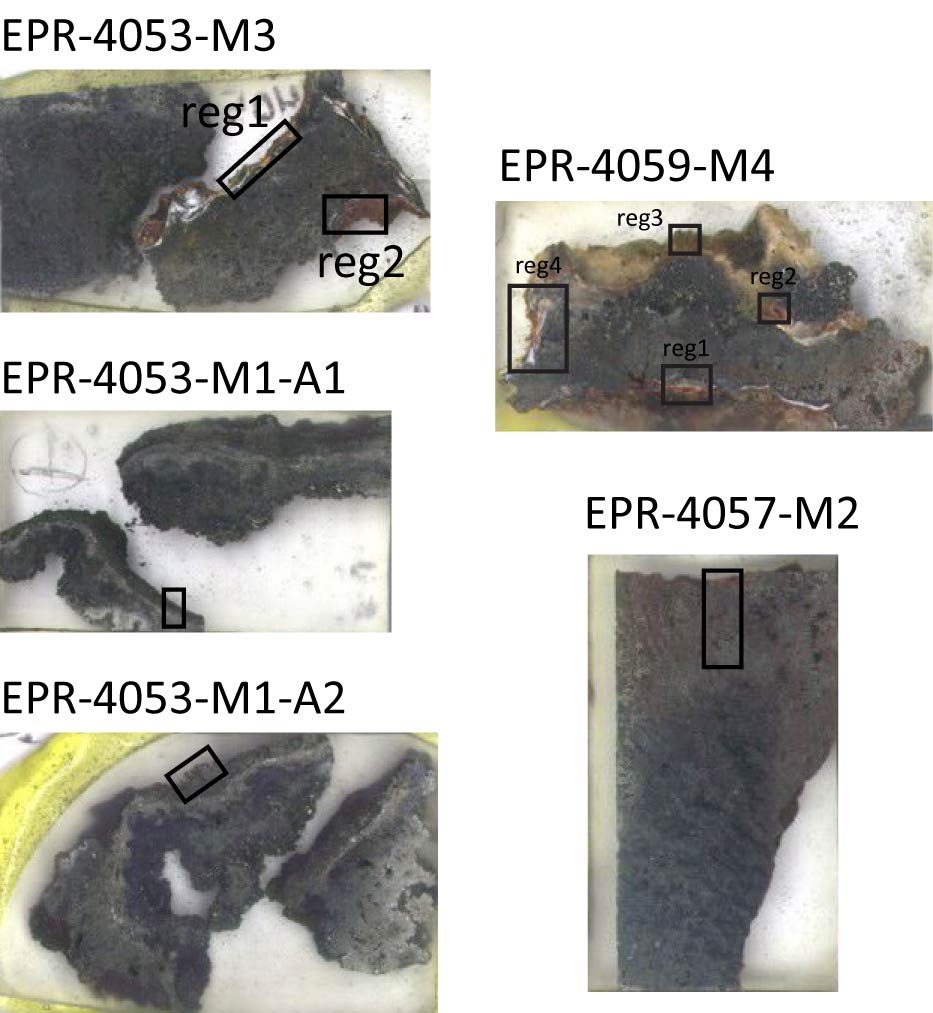

Supplement: Figure S4 — Photographs of polished thick sections showing the regions of the sample investigated. [file Image4.JPEG]

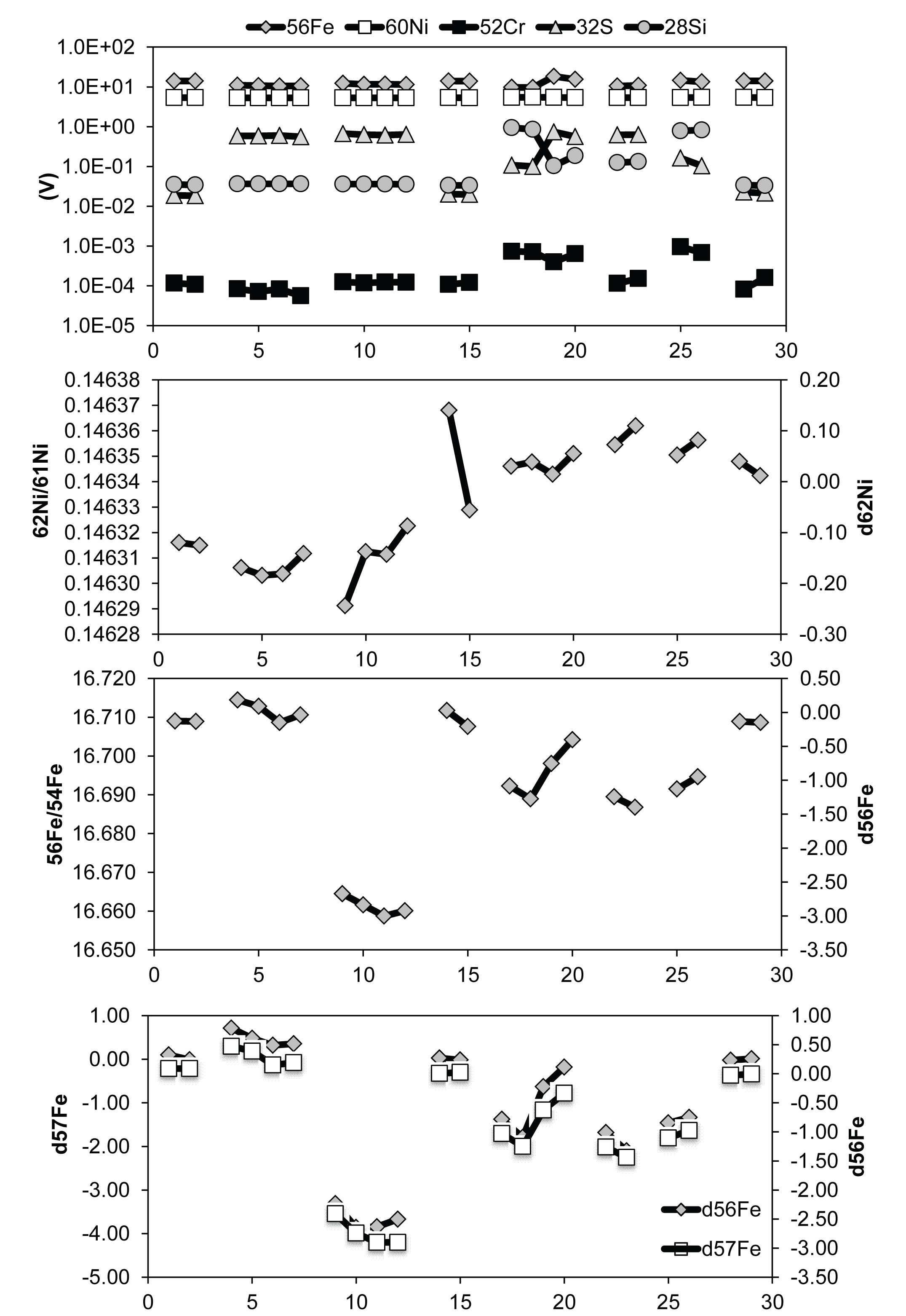

Supplement: Figure S5 — Example laser ablation run. [file Image5.JPEG]

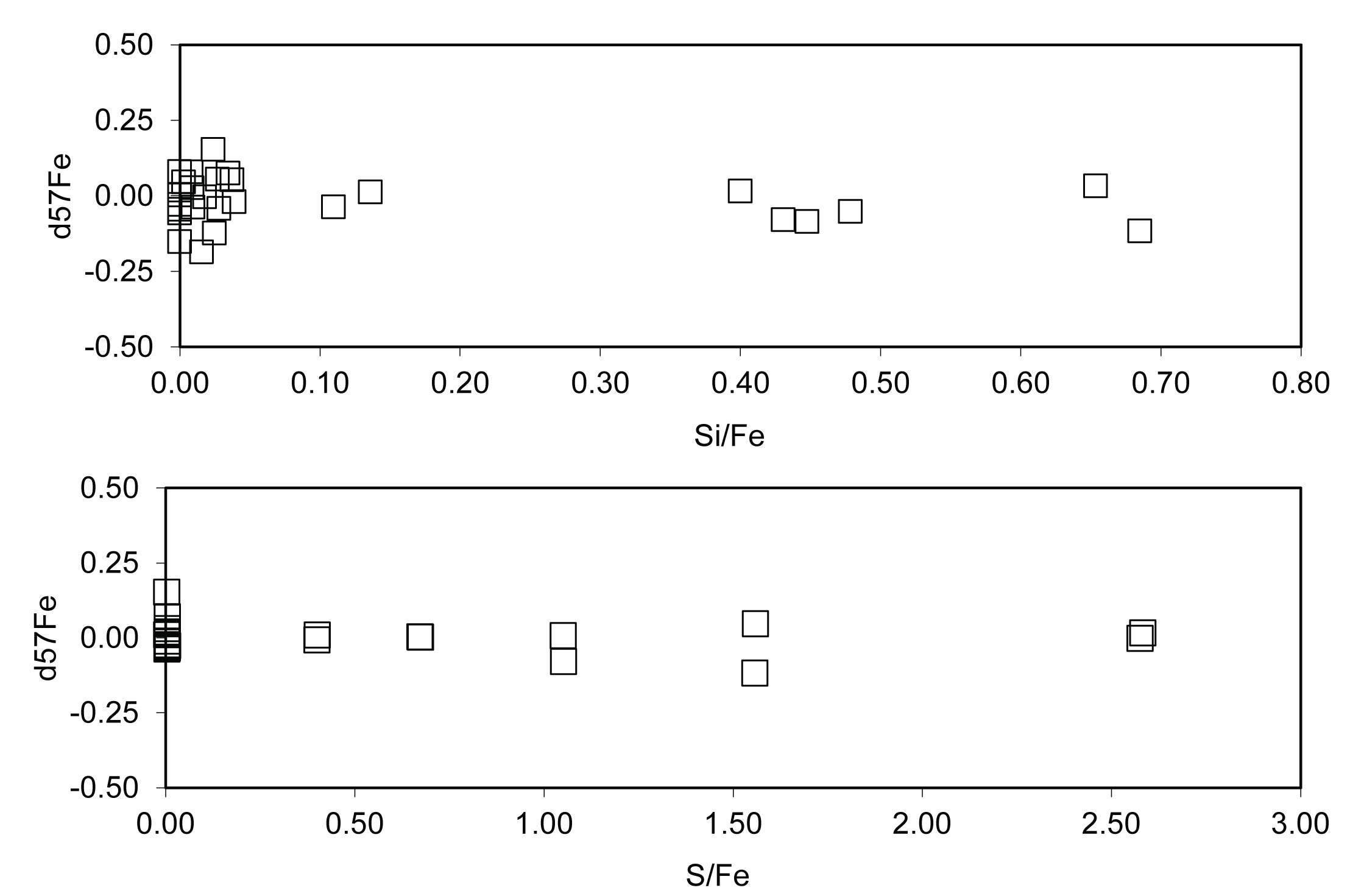

Supplement: Figure S6 — Iron isotope values as a function of S:Fe and Si:Fe. [file Image6.JPEG]

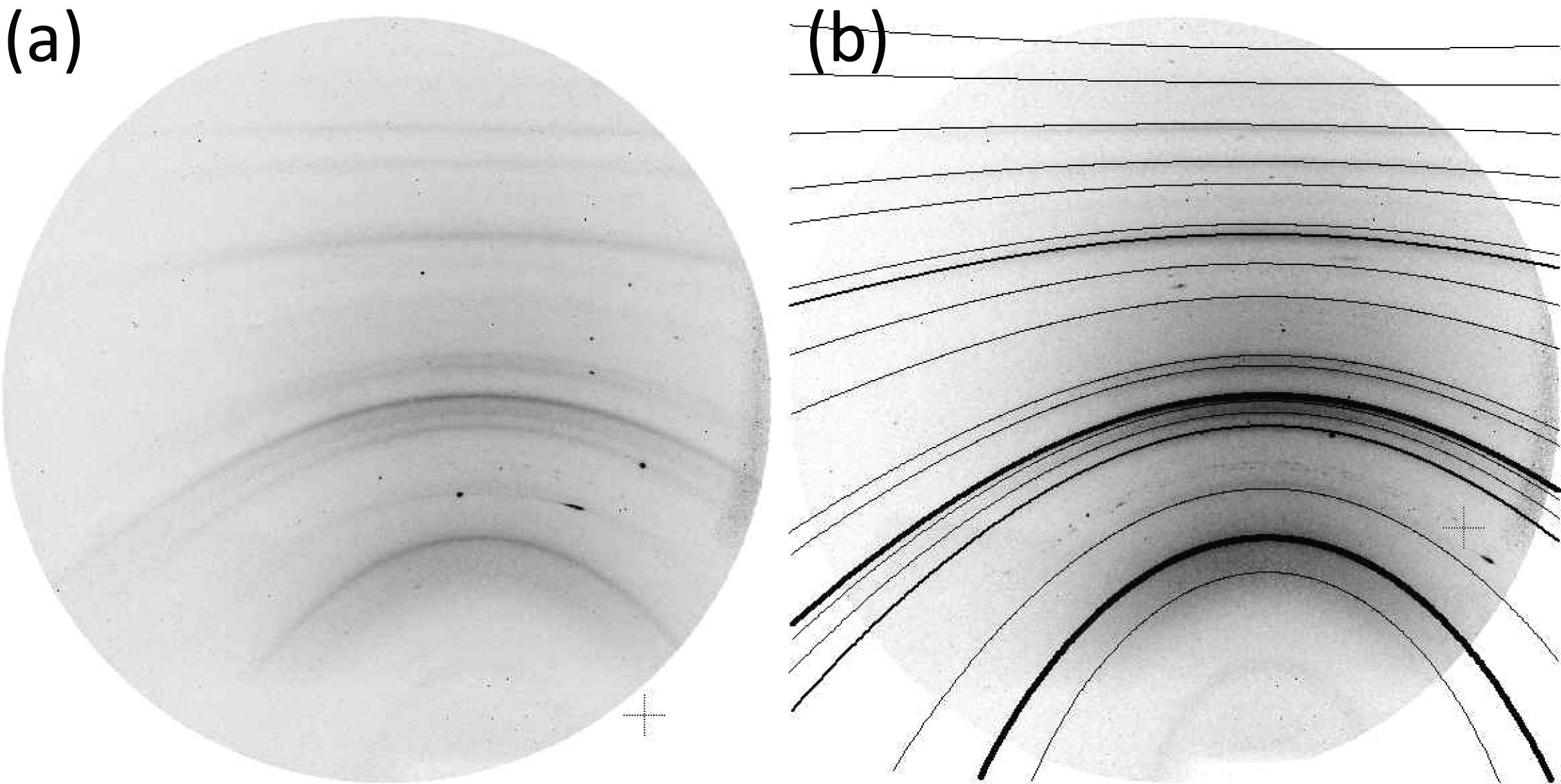

Supplement: Figure S7 — (A) Synchrotron radiation microprobe X-ray diffraction data from spot 2 of EPR-4057-M2. (B) Data with goethite reference lines. [file Image7.JPEG]

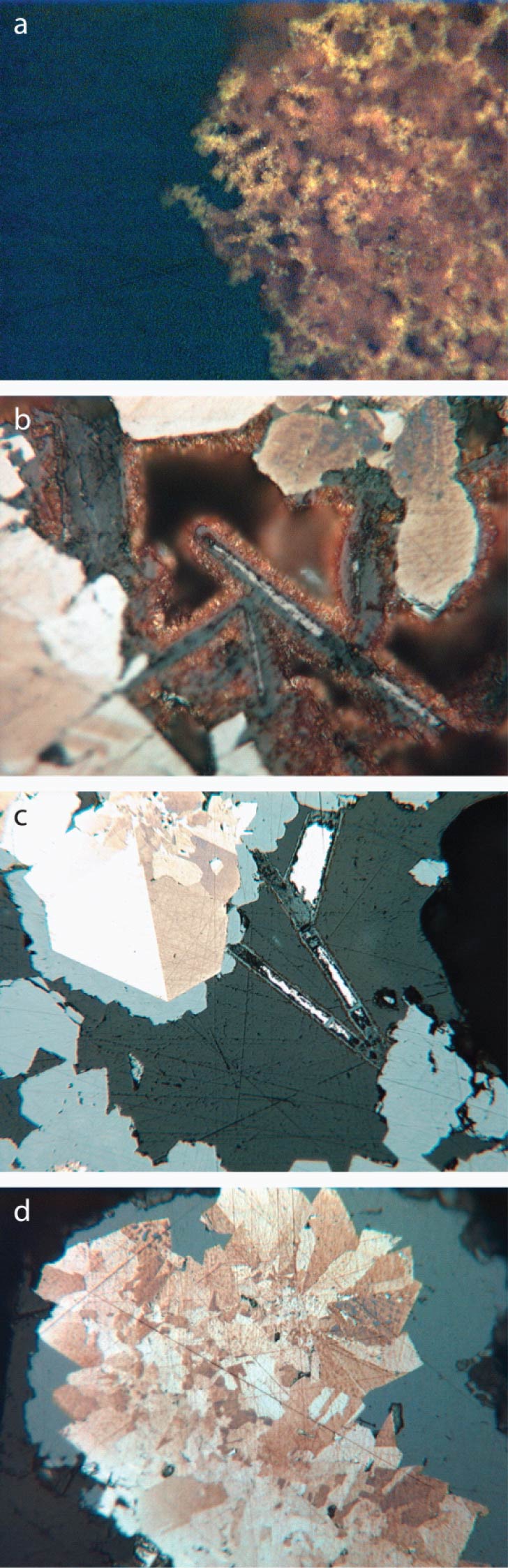

Supplement: Figure S8 — Petrographic microscope images of sample EPR-4057-M2. (A) Iron oxyhydroxide accumulations at the seawater exposed surface of the massive sulfide deposit. (B) Oxidation of pyrrhotite crystals. (C,D) Sulfide minerals. [file Image8.JPEG]

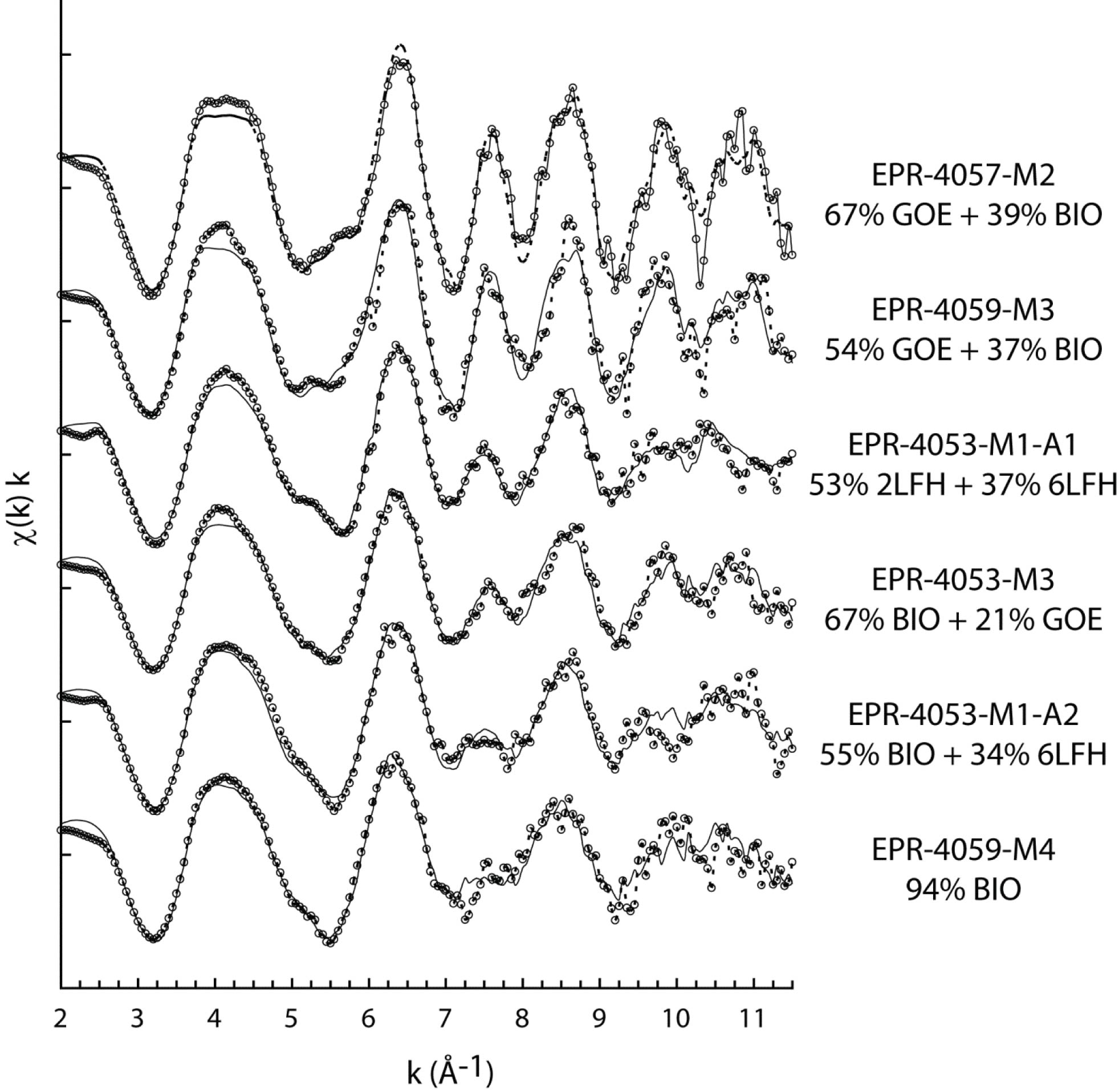

Supplement: Figure S9 — Summary of Fe EXAFS data (dots) and best linear combination fit (solid lines). Goodness of fit parameters (Table S4) and key for reference materials (Table S2) are provided in the main text. [file Image9.JPEG]
