# Supplementary material for: Iron Transformation Pathways and Redox Micro-Environments in Seafloor Sulfide-Mineral Deposits: Spatially Resolved Fe XAS and δ57/54Fe Observations
Source: Front Microbiol. 2016 May 10;7:648. doi: 10.3389/fmicb.2016.00648 (PMC4862312; doi:10.3389/fmicb.2016.00648)
Supplement: Supplementary file 11 [file DataSheet1.DOCX]

**Supplemental**

**Iron transformation pathways and redox micro-environments in seafloor**

**sulfide-mineral deposits: spatially resolved Fe XAS and δ^57/54^Fe observations**

Brandy M. Toner^1*^, Olivier Rouxel^2^, Cara M. Santelli^3^, Wolfgang Bach^4^, and Katrina J. Edwards^5,†^

**
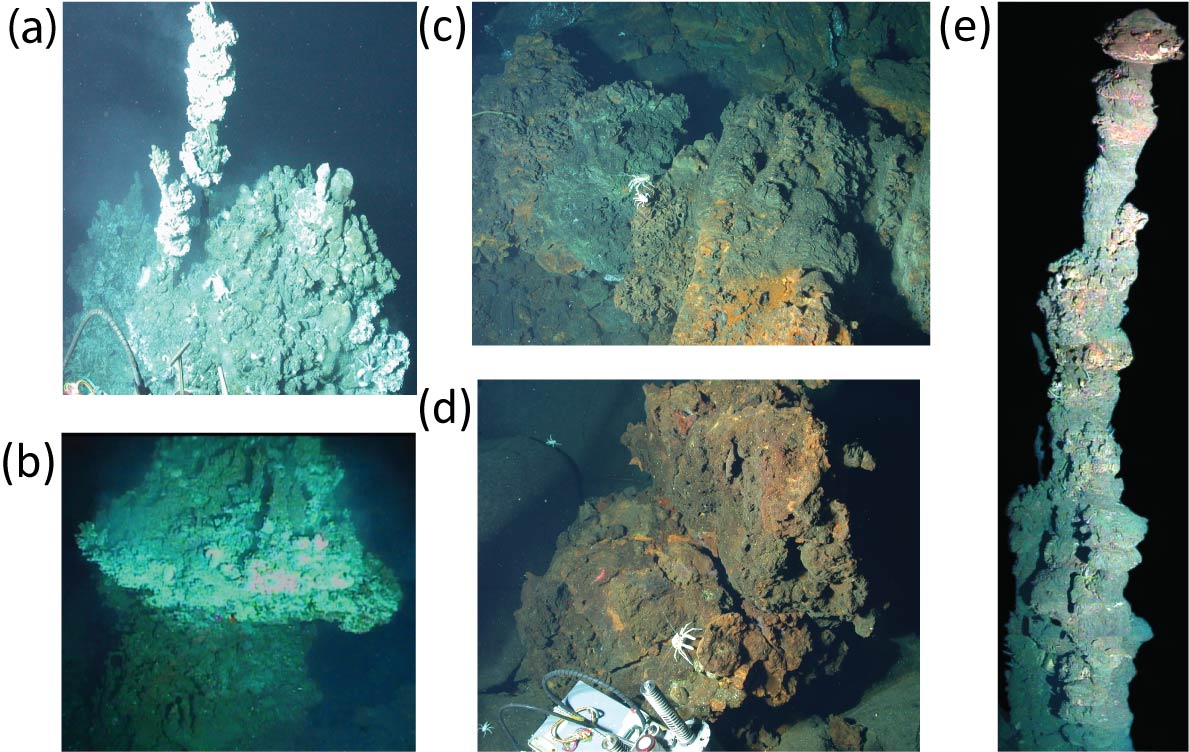
**

**Figure S1.** Seafloor photographs of sampling sites. a, b) Location of K Vent samples EPR-

4053-M2, EPR-4053-M1-A1, and EPR-4053-M1-A2. c, d) Massive sulfide deposits in the Bio9 Vent area EPR-4057-M2 and EPR-4059-M3, respectively. e) Off axis, extinct chimney sources of sample EPR-4059-M4. Images previously published in various forms in Rouxel et al. 2008, Sylvan et al. 2012, and Toner et al. 2013.

*Figure S1.jpeg (RGB 300 dpi)*

**
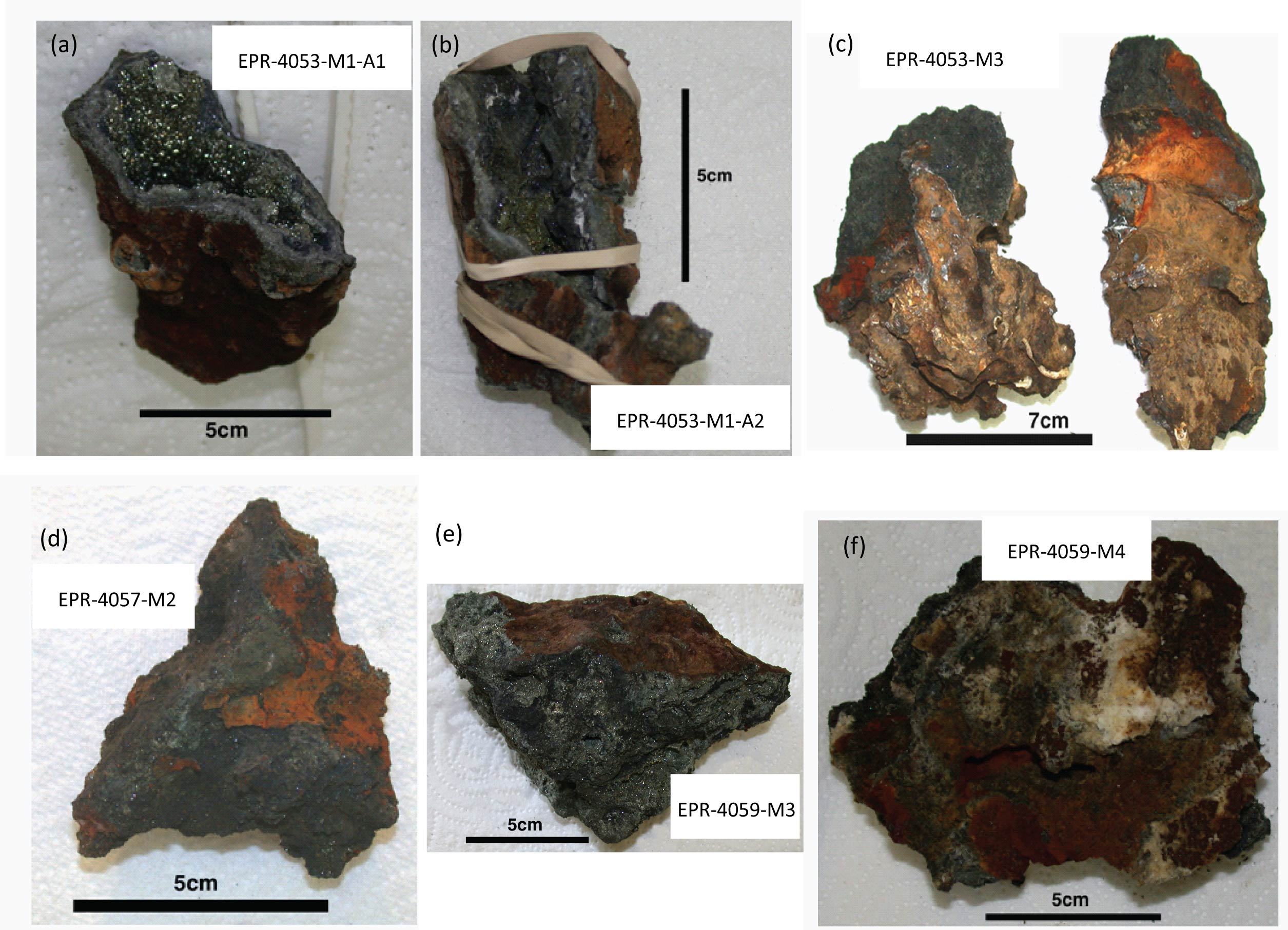
**

**Figure S2.** Shipboard photographs of samples.

*Figure S2.jpeg (RGB 300 dpi)*

**
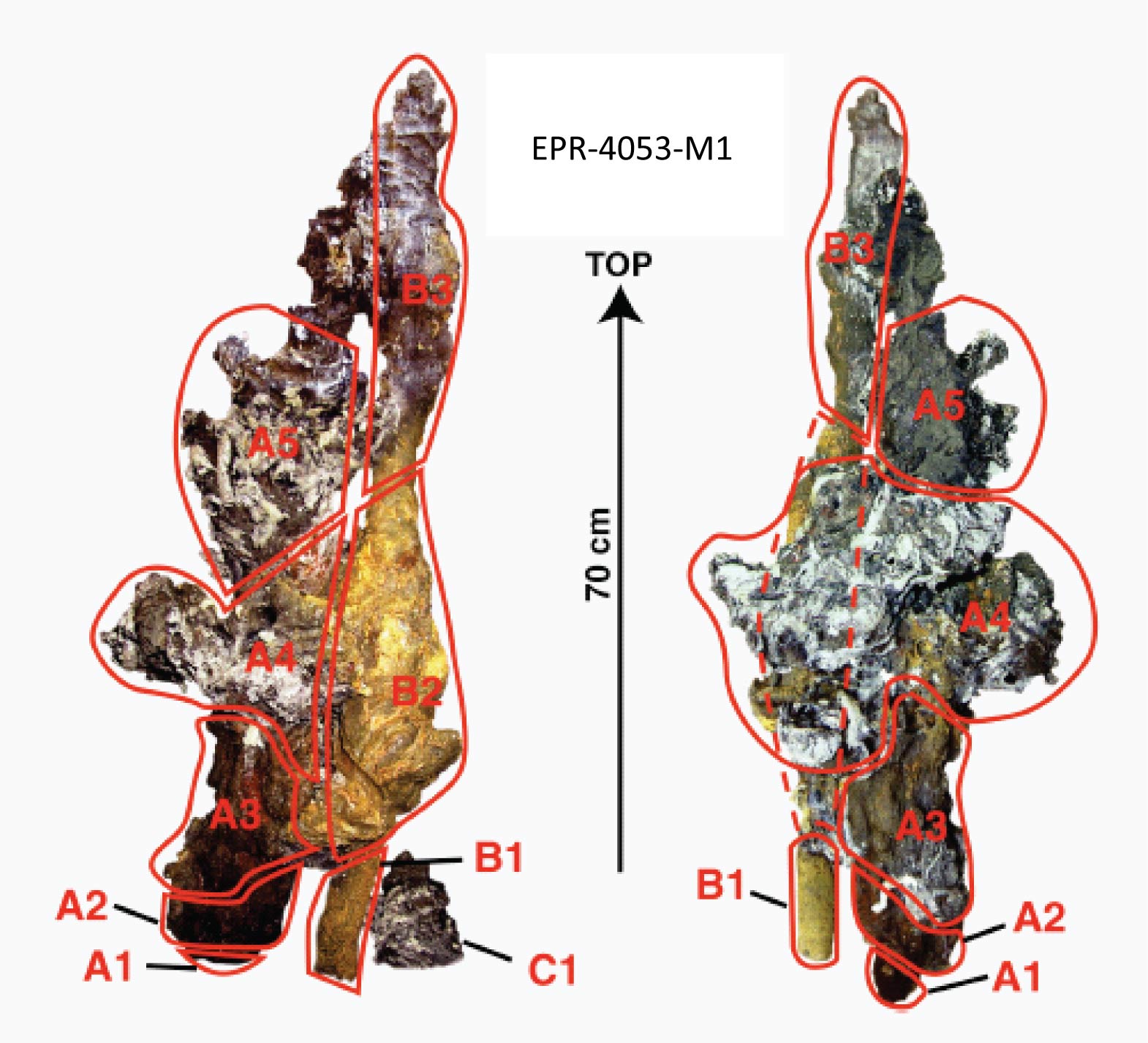
**

**Figure S3.** Shipboard photographs of K Vent inactive spire showing the locations of EPR-4053-M1-A1 and EPR-4053-M1-A2. Portions of this figure are published in Rouxel et al. 2008.

*Figure S3.jpeg (RGB 300 dpi)*

**
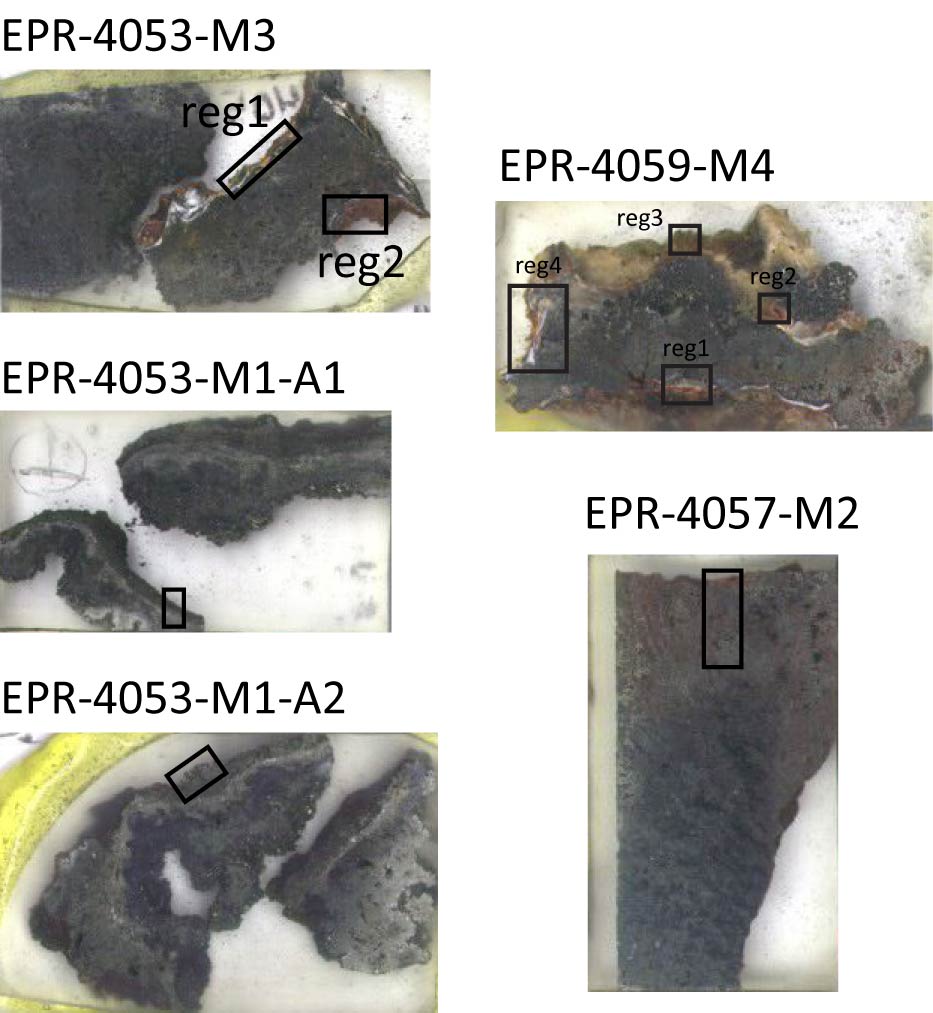
**

**Figure S4.** Photographs of polished thick sections showing the regions of the sample investigated.

*Figure S4.jpeg (RGB 300 dpi)*

**
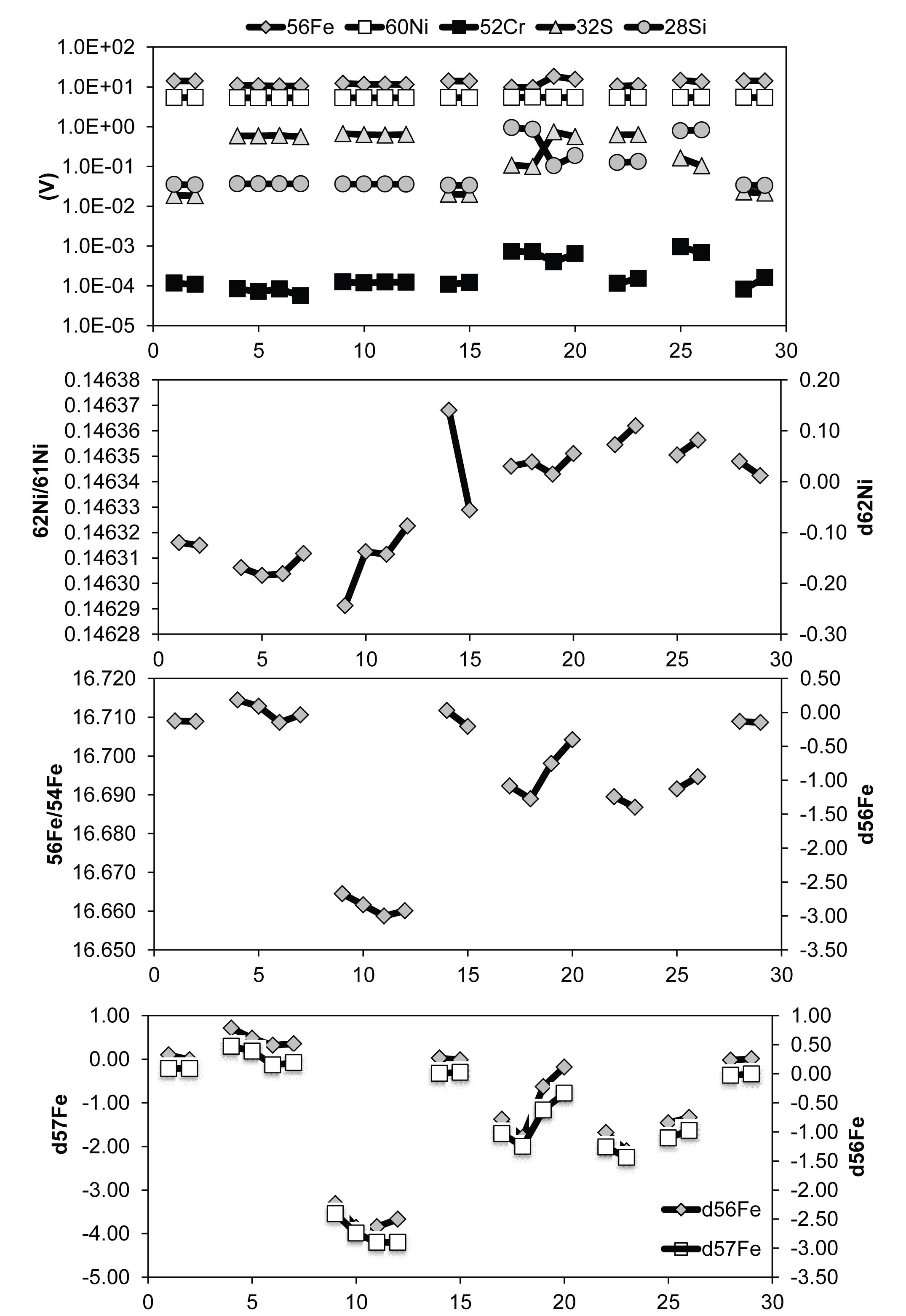
**

**Figure S5.** Example laser ablation run.

*Figure S5.jpeg (RGB 300 dpi)*

**
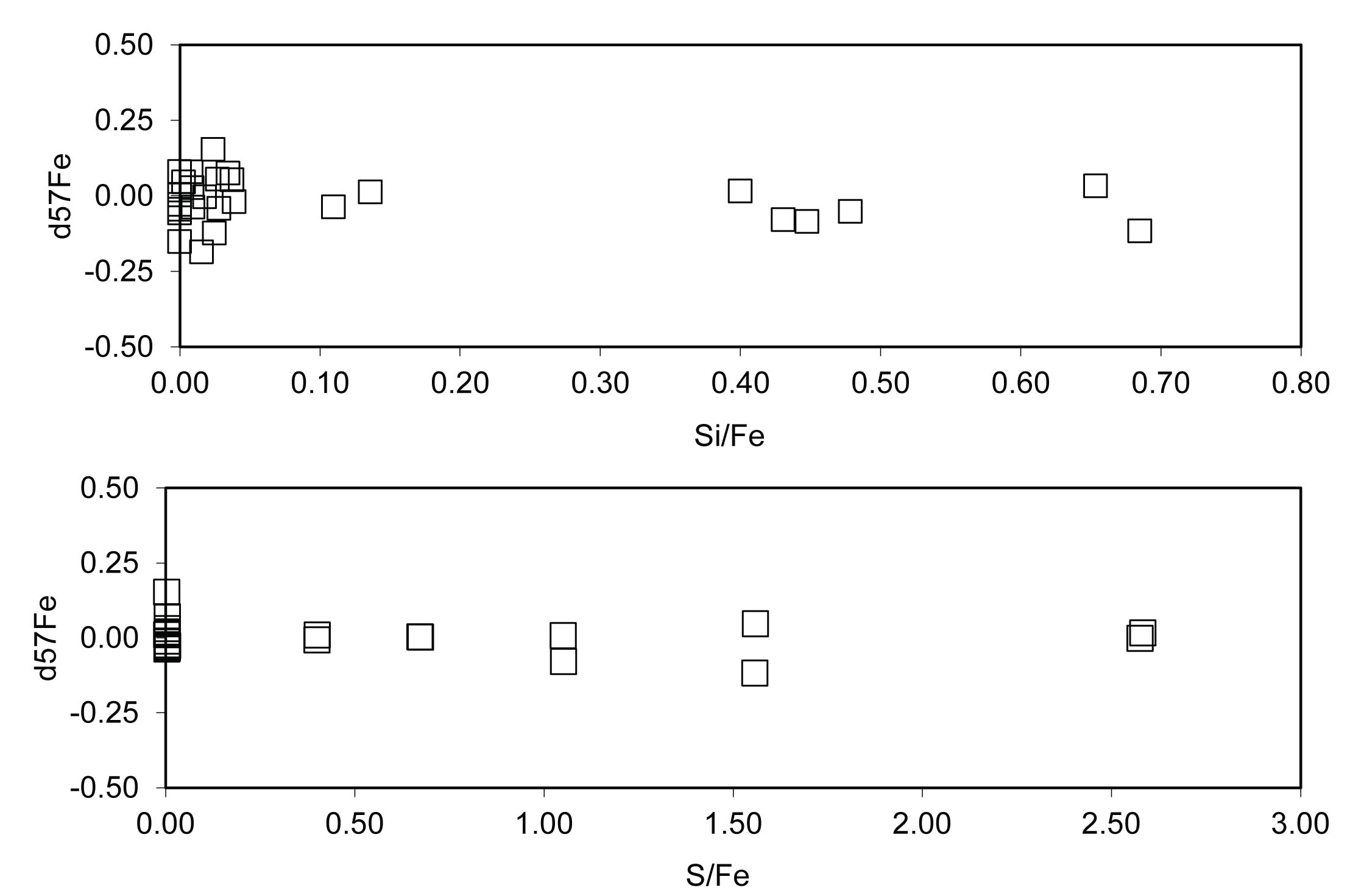
**

**Figure S6.** Iron isotope values as a function of S:Fe.

*Figure S6.jpeg (RGB 300 dpi)*

**
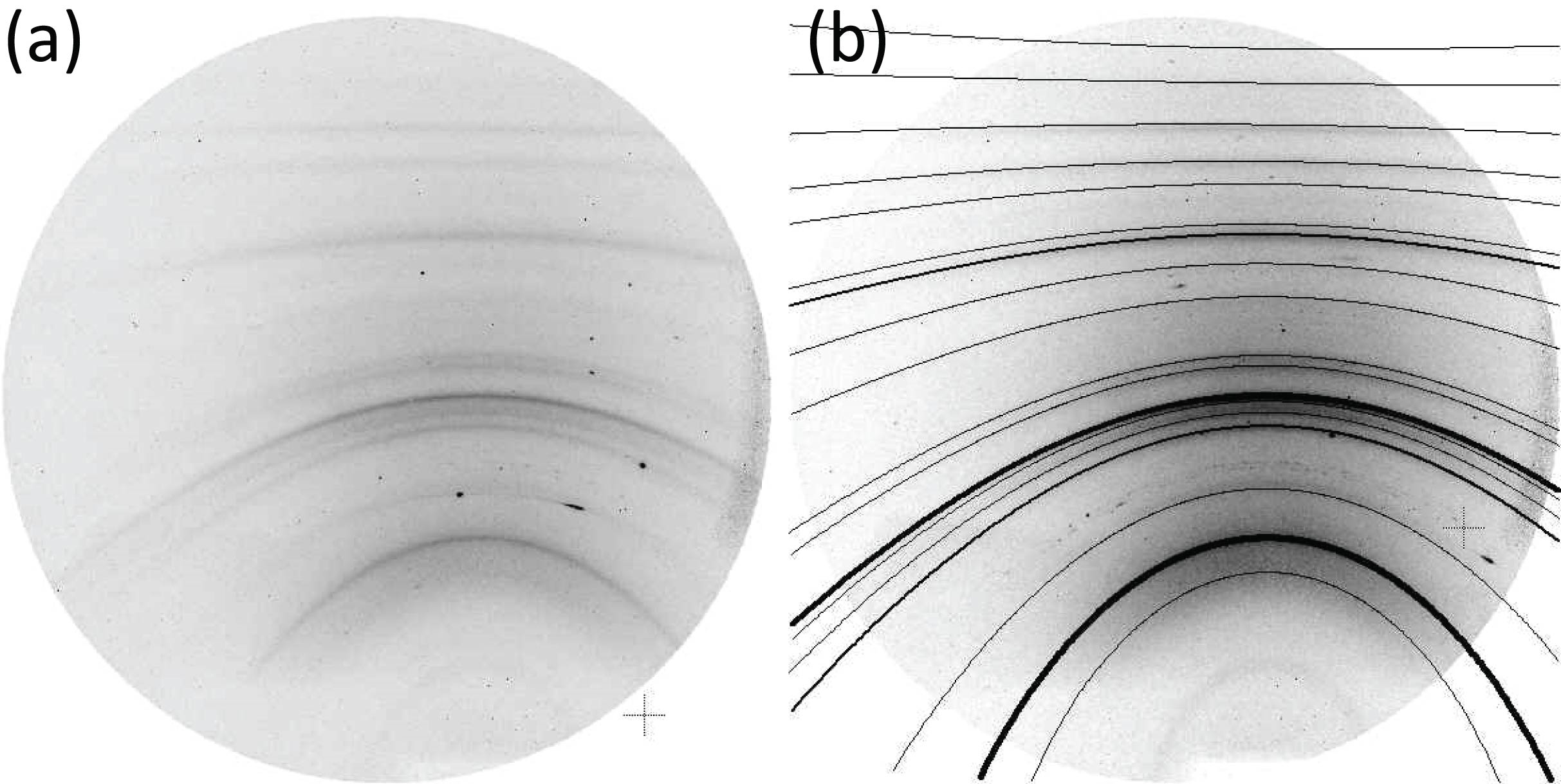
**

**Figure S7.** a) Synchrotron radiation microprobe X-ray diffraction data from spot 2 of EPR-4057-M2. b) Data with goethite reference lines.

*Figure S7.jpeg (RGB 300 dpi)*

**
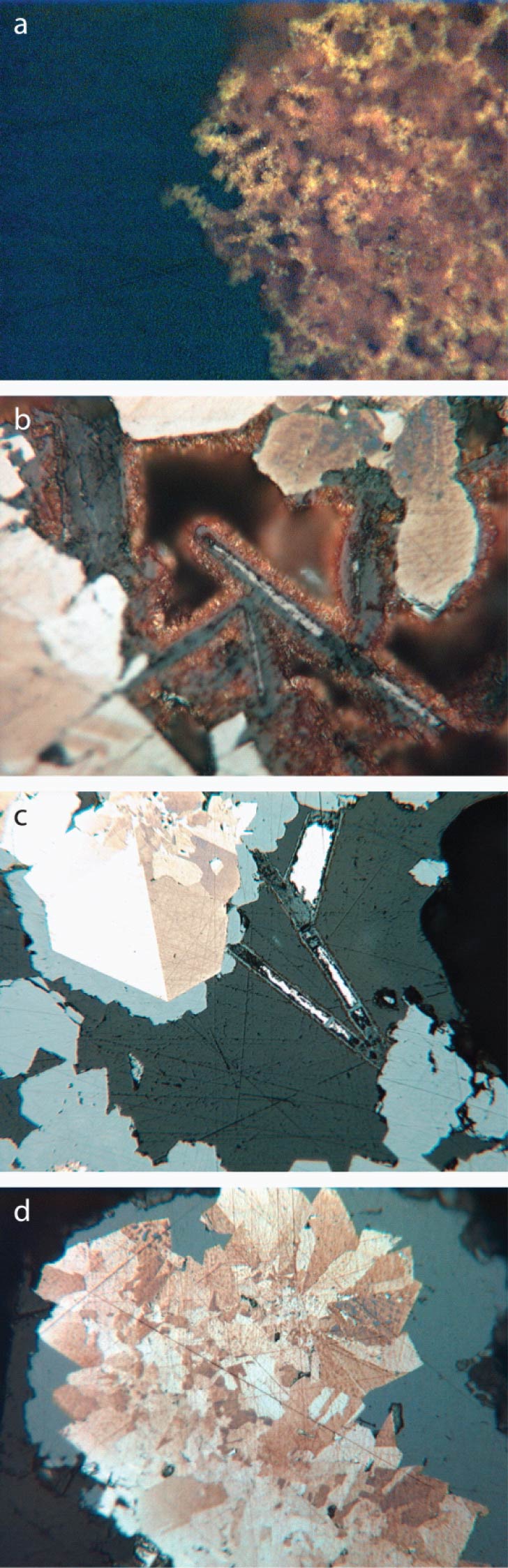
**

**Figure S8.** Petrographic microscope images of sample EPR-4057-M2. a) Iron oxyhydroxide accumulations at the seawater exposed surface of the massive sulfide deposit. b) Oxidation of pyrrhotite crystals. c,d) Sulfide minerals.*Figure S8.jpeg (RGB 300 dpi)*

**
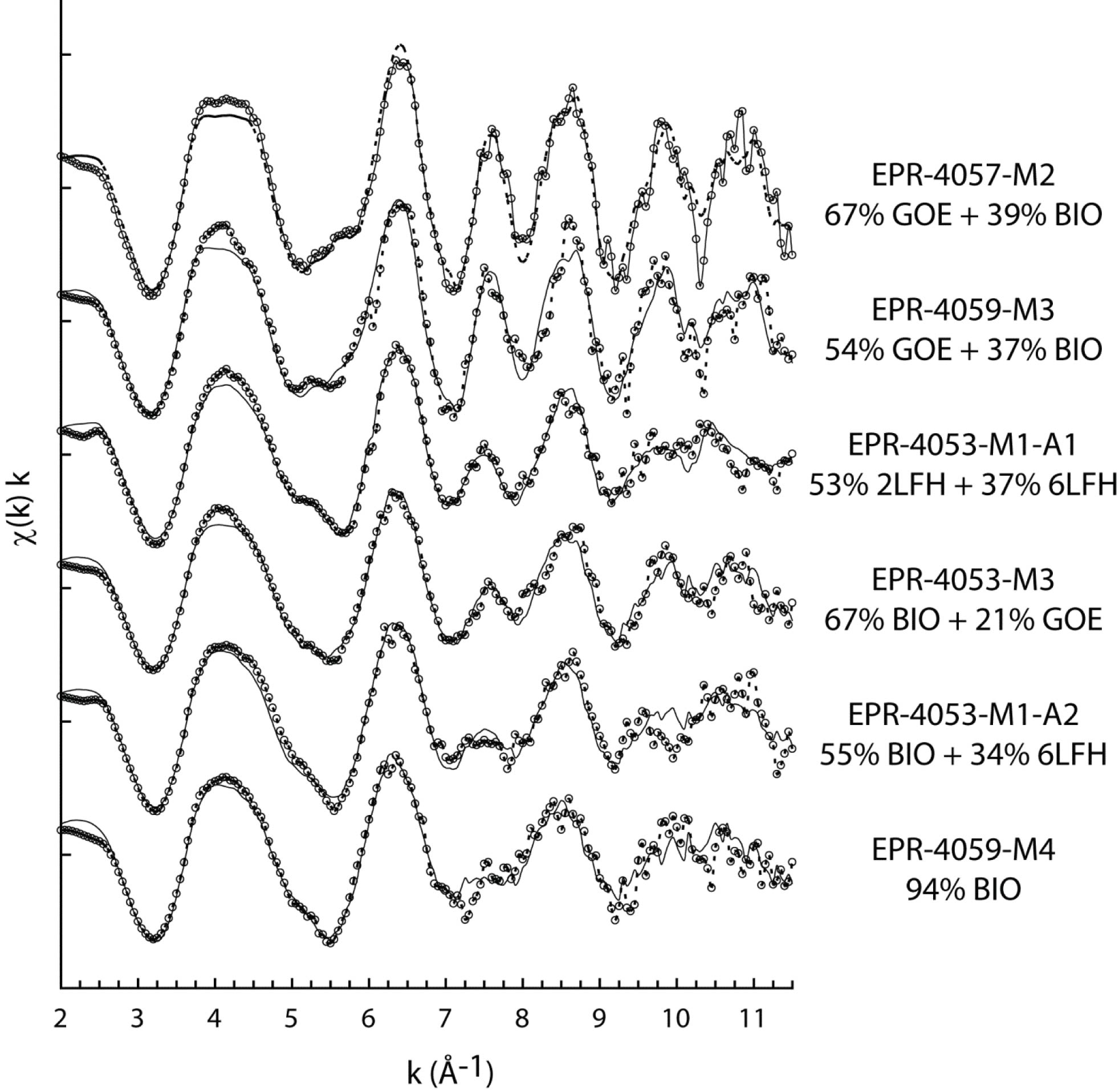
**

**Figure S9.** Summary of Fe EXAFS data (dots) and best linear combination fit (solid lines). Goodness of fit parameters (Table 4) and key for reference materials (Table 2) are provided in the main text.

*Figure S9.jpeg (RGB 300 dpi)*
